# Vasoactive–Inotropic Score Reduction Rate Is Highly Associated With Prognosis for Critically Ill Patients With Cardiogenic Shock: Insights From the Real‐World Dynamic Data

**DOI:** 10.1155/cdr/8989505

**Published:** 2026-03-06

**Authors:** Yi-Le Ning, Tian-Xiang Guan, Xiao-Li Niu, Qian-Qian Ma, Yuan-Na Zhang, Ting-Yu Peng, Li-Xiong Zeng, Hang Li, Hui-Ting Guan

**Affiliations:** ^1^ Department of Critical Care Medicine, Shenzhen Bao′an Chinese Medicine Hospital, Shenzhen, China; ^2^ The Fourth Clinical College, Guangzhou University of Chinese Medicine, Shenzhen, China, gzucm.edu.cn; ^3^ The Seventh Clinical College, Guangzhou University of Chinese Medicine, Shenzhen, China, gzucm.edu.cn; ^4^ Department of Cardiology, The Third Xiangya Hospital of Central South University, Changsha, China, csu.edu.cn; ^5^ Department of Central Lab, Shenzhen Bao′an Chinese Medicine Hospital, Shenzhen, China; ^6^ Department of Rehabilitation, Shenzhen Bao′an Chinese Medicine Hospital, Shenzhen, China

**Keywords:** cardiogenic shock, hemodynamic support, mortality, vasoactive–inotropic score, VIS reduction rate

## Abstract

**Background:**

In cardiogenic shock (CS), titration of both the choice and dosage of inotropes and vasopressors is crucial. The vasoactive–inotropic score (VIS) quantifies hemodynamic support, while the effect of temporal VIS change on prognosis remains unclear. This study evaluated the association between the vasoactive–inotropic score reduction rate (VRR) to measure VIS changes over time and explore its association with mortality in CS patients.

**Methods:**

We performed a retrospective observational study using two large intensive care databases (MIMIC‐IV and eICU). Adult patients with CS receiving vasoactive–inotropic agents within 24 h after ICU admission were included. VIS was calculated using the updated VIS 2020, and VRR was defined to capture the relative change in VIS over time. Patients were categorized into VIS‐decreasing and VIS‐increasing groups. The primary outcome was in‐hospital mortality; secondary outcomes were intensive care unit (ICU) and 28‐day mortality (28‐day mortality only for the MIMIC‐IV database). Associations between VRR and outcomes were examined using four models: (1) log‐rank analysis, (2) Cox model adjusted for all covariates, (3) Cox model adjusted for covariates selected by univariable analyses, and (4) Cox model adjusted for covariates selected by a random forest algorithm.

**Results:**

A total of 3170 adult CS patients were analyzed. All models in both MIMIC‐IV and eICU showed that an increasing VIS over time was consistently associated with higher in‐hospital, ICU, and 28‐day mortality compared with a decreasing VIS in the MIMIC‐IV cohort (*p* < 0.001). These findings were reproduced in the external eICU cohort for ICU and in‐hospital mortality (*p* < 0.001), supporting the robustness and generalizability of VRR as a prognostic indicator across heterogeneous ICUs.

**Conclusions:**

In adult CS patients, a greater reduction in VIS over time is strongly associated with lower mortality. VRR provides a simple, dynamic summary of vasoactive trajectories and may serve as a useful adjunct for risk stratification alongside other clinical and biochemical markers, although prospective validation is warranted.

## 1. Introduction

Cardiogenic shock (CS) is a critical clinical condition characterized by acute hypoperfusion and organ dysfunction due to a significant decrease in cardiac output (CO), which is commonly caused by acute myocardial infarction (AMI) or left ventricular dysfunction [1–3]. The primary treatment for CS involves the use of vasoactive agents, such as vasopressors and inotropes, which improve end‐organ perfusion by increasing CO and blood pressure (BP), thereby preventing multiorgan failure [4]. However, while these medications are widely used in clinical practice, current guidelines do not provide explicit recommendations on which specific drugs or combinations to use, mainly due to patient variability and the differing effects of these drugs at various stages of illness.

Although vasoactive–inotropic agents play a crucial role in the management of CS, their use is not without risks. Studies have indicated that the administration of drugs such as dopamine and norepinephrine is associated with an increased risk of mortality and arrhythmias [5, 6]. For instance, norepinephrine use has been linked to a higher incidence of myocardial infarction and peripheral ischemia, while dopamine is associated with a greater occurrence of cardiac arrhythmias. Additionally, epinephrine and terlipressin have also been implicated in increasing the risks of myocardial infarction and peripheral ischemia [7]. However, these findings are not universally applicable, as the potential benefits of vasoactive agents may outweigh the risks in certain CS patient populations. Therefore, assessing these adverse effects requires careful consideration of individual patient characteristics and clinical context.

Current research on the impact of dosing and timing trends of vasoactive–inotropic agents on mortality in CS patients is still limited. Although some recent studies have begun to investigate how different doses and durations of drug administration affect hemodynamic management after cardiac surgery or in shock treatment strategies, these studies often focus on specific types of shock (e.g., septic shock). There is still a need for more comprehensive research into how the use of different vasoactive agents affects outcomes in CS, particularly concerning their dosage trends and time‐related effects [8]. Understanding the impact of these factors on the prognosis of CS patients is therefore of great clinical importance.

The degree of hemodynamic support provided by vasoactive agents is commonly considered an indicator of disease severity in critically ill patients, with higher drug requirements often predicting a greater risk of mortality [9]. However, the clinical practice of administering vasoactive–inotropic agents varies significantly among physicians and healthcare institutions. The vasoactive–inotropic score (VIS) is a method of quantifying hemodynamic support by calculating the weighted doses of all administered inotropic and vasoconstrictive agents. This objective approach standardizes the dosing of different drugs and serves as an effective prognostic tool [10]. VIS has been applied to various clinical contexts, including predicting mortality and morbidity in cardiac surgery, pediatric surgery, septic shock, and extracorporeal membrane oxygenation (ECMO) patients [11–13]. This study adopts the updated 2020 version of VIS, which includes additional agents such as enoximone, levosimendan, olprinone, methylene blue, phenylephrine, terlipressin, and angiotensin II. Nevertheless, the relationship between temporal changes in VIS and clinical outcomes in adult CS patients remains unexplored.

Accordingly, we adopted the vasoactive–inotropic score reduction rate (VRR) to quantify changes in vasoactive agent use during the early phase following intensive care unit (ICU) admission. We aimed to explore the association between early decreases in VIS and prognosis in CS patients, providing new insights into the clinical management of CS.

## 2. Methods

### 2.1. Study Population

Based on the MIMIC‐IV database (Version 3.1) and the eICU Collaborative Research Database (Version 2.0), this study focused on adult patients with CS. MIMIC‐IV, maintained by the Massachusetts Institute of Technology Laboratory for Computational Physiology and collaborating research groups, contains hourly high‐resolution monitoring data and comprehensive electronic medical records for ICU patients. The eICU database is a multicenter ICU database including data from over 200 hospitals across the United States, with detailed information on physiologic parameters, treatments, and outcomes. Our research team obtained the necessary authorization to access the MIMIC‐IV and eICU databases (Authorization ID: 40974208). Patients with CS were first identified using International Classification of Diseases (ICD) codes in the MIMIC‐IV and eICU databases. From this initial cohort, we excluded patients according to the flowchart‐based process: (1) those in whom vasopressors were not initiated, or were initiated more than 24 h after ICU admission, (2) those with ICU length of stay ≤ 24 h, (3) patients younger than 18 years, and (4) cases in which survival time or outcome information was incorrect or unavailable.

### 2.2. VIS and VRR

The 2020 version of the VIS formula is as follows [10]: VIS = 10,000 × vasopressin dose (U/kg/min/) + 100 × epinephrine dose (*μ*g/kg/min)+ 100 × norepinephrine dose (*μ*g/kg/min) + 50 × levosimendan dose (*μ*g/kg/min) + 25 × olprinone dose (*μ*g/kg/min) + 20 × methylene blue dose (mg/kg/h) + 10 × milrinone dose (*μ*g/kg/min + 10 × phenylephrine dose (*μ*g/kg/min) + 10 × terlipressin dose (*μ*g/min) + 0.25 × angiotensin II dose (ng/kg/min) + dopamine dose (*μ*g/kg/min) + dobutamine dose (*μ*g/kg/min) + enoximone dose (*μ*g/kg/min).

Compared to the 2010 version, VIS 2020 has incorporated additional agents, including enoximone, levosimendan, olprinone, methylene blue, phenylephrine, terlipressin, and angiotensin II. Due to the tradition of hospitals and physicians using vasoactive drugs, we found most of the vasopressors (dopamine, dobutamine, epinephrine, milrinone, vasopressin, norepinephrine, and phenylephrine) in the VIS 2020 formula in the MIMIC‐IV database and constructed a real‐time information matrix of multiple vasoactive drugs based on the patient′s drug infusion rates and timepoints information.

VRR is a concept we cited to quantify dosage reduction for vasoactive–inotropic agents. The formula of VRR is defined as follows: (VIS_1−24h max_–VIS_25−48h max_)/VIS_1−24h max_.

### 2.3. Data Extraction

The real‐time VIS was calculated according to the infusion rates of commonly used vasoactive and inotropic agents, including dopamine, dobutamine, epinephrine, milrinone, vasopressin, norepinephrine, phenylephrine, terlipressin, angiotensin II, levosimendan, olprinone, methylene blue, and enoximone. Although the specific vasoactive/inotropic agents available in the MIMIC‐IV and eICU databases are not identical, both datasets capture the majority of drug classes incorporated in the contemporary VIS framework. In the MIMIC‐IV database, recorded vasoactive/inotropic agents included vasopressin, dopamine, dobutamine, epinephrine, milrinone, norepinephrine, phenylephrine, and angiotensin II. In the eICU database, the available agents comprised vasopressin, dopamine, dobutamine, epinephrine, methylene blue, milrinone, norepinephrine, and phenylephrine. Consequently, while a few less frequently used agents (such as levosimendan and olprinone) could not be incorporated due to their absence or very low prevalence in these databases, the principal components of vasoactive support in contemporary adult intensive care practice were well represented. We therefore considered the calculated VIS in MIMIC‐IV and eICU to provide a reasonably comprehensive and comparable quantification of the overall vasoactive/inotropic burden across both cohorts.

Our research database consolidated diverse clinical parameters into a unified matrix. The collected data spanned patient demographics, admission records, and disease severity metrics. We integrated vital signs monitoring, laboratory analyses, and therapeutic protocols. Standardized evaluation systems, specifically the Simplified Acute Physiology Score II (SAPS II) and Sequential Organ Failure Assessment (SOFA) score, were incorporated to assess patient status. The database also captured comorbidity patterns and clinical endpoints.

### 2.4. Outcome

The primary outcome was in‐hospital mortality. ICU mortality and 28‐day mortality (the latter assessed only in the MIMIC‐IV database) were defined as secondary outcomes.

### 2.5. Covariates

In this investigation, we classified 37 variables into eight distinct domains as covariates. The demographic parameters encompassed age, gender, ethnicity, and body weight. Clinical severity was assessed through standardized indices, including SAPS II, SOFA score, and the Charlson comorbidity index. Therapeutic interventions comprise mechanical ventilation, administration of sedation, and albumin. Concurrent medical conditions were extensively tracked, including heart failure, hypertension, atrial fibrillation, Type 2 diabetes mellitus, chronic kidney disease, chronic liver disease, chronic obstructive pulmonary disease (COPD), coronary artery disease, cerebrovascular events, and malignancies. Physiological parameters focused on heart rate, mean arterial pressure (MAP), and body temperature. Laboratory assessments incorporated leukocyte count, hemoglobin level, platelet count, pH, partial pressures of oxygen and carbon dioxide, lactate concentration, and creatinine level. For statistical rigor, variables with missing data exceeding 40% were excluded from the covariate analysis model [14].

### 2.6. Interpretable Machine Learning Models and Statistical Analysis

We developed prediction models for in‐hospital mortality to evaluate the predictive performance of VRR. The MIMIC‐IV dataset was randomly split into a training set and an internal validation set in a 7:3 ratio, and the eICU dataset was used as an independent external validation cohort. Candidate features were selected with LASSO regression. We evaluated 12 supervised learning algorithms: random forest, XGBoost, LightGBM, CatBoost, gradient boosting, AdaBoost, extra trees, logistic regression, support vector machine (SVM), SGD classifier, multilayer perceptron (neural network), and Gaussian Naive Bayes. Each algorithm had a predefined hyperparameter grid (e.g., number of trees, maximum depth, learning rate, and number of leaves). We performed 5‐fold cross‐validation with GridSearchCV using the area under the receiver operator characteristic (ROC) curve (AUC) as the primary optimization metric. Accuracy, sensitivity (recall), precision, and *F*1‐score were also computed. For each model, the pipeline with the highest cross‐validated AUC was selected and saved for downstream analysis. On the internal and external validation sets, the model output predicted probabilities for in‐hospital mortality. We then calculated AUC, accuracy, sensitivity, specificity, positive and negative predictive values, false‐positive and false‐negative rates, balanced accuracy, F‐beta (*F*1) score, cross‐entropy loss, diagnostic odds ratio, and Matthews correlation coefficient. ROC curves were plotted, and AUCs with 95% confidence intervals (CIs) were summarized for comparison of algorithms. To improve clinical interpretability, we applied SHapley Additive exPlanations (SHAP) to tree‐based models (random forest, XGBoost, LightGBM, CatBoost, gradient boosting, and AdaBoost). For each model, we reloaded the trained pipeline, randomly sampled up to 500 test‐set patients, applied the preprocessing steps, and used SHAP. TreeExplainer was used to compute SHAP values for the positive class. SHAP summary dot plots and bar plots rank features by mean SHAP value, describing the overall impact of each predictor on the model output.

Data normality assessment followed a dual approach: the Shapiro–Wilk test was utilized for samples of 2000 or fewer observations, while the Kolmogorov–Smirnov test was applied to larger datasets. Between‐group variance equality was evaluated using Levene′s test. The reporting format for continuous variables was distribution‐dependent: mean (standard deviation) for normal distributions and median (interquartile range) for non‐normal distributions. We applied *t*‐tests to compare continuous variables when normality and variance homogeneity assumptions were met; otherwise, Wilcoxon′s rank‐sum tests were used. For categorical variables, expressed as counts (percentages), we employed chi‐square tests, defaulting to Fisher′s exact test when the sample size was less than 40.

We conducted survival analyses using Kaplan–Meier methodology and compared survival between groups using log‐rank tests. Hazard ratios (HRs) and 95% CIs for the association between VRR categories and time‐to‐event outcomes were further estimated using multivariable Cox proportional hazards models under four predefined strategies. Model 1 was an unadjusted log‐rank model, from which HRs (95% CIs) were derived based on Kaplan–Meier curves. Model 2 was a multivariable Cox model adjusted for all candidate covariates considered clinically relevant or previously reported to be associated with prognosis in CS. Model 3 was a multivariable Cox model adjusted for covariates that were significantly associated with the outcome in univariable analyses. Model 4 was a multivariable Cox model adjusted for covariates selected by a random forest algorithm, which was used to identify the most important predictors based on variable importance measures.

Machine learning models were conducted using Python 3.12.12 on Ubuntu LTS 22.04. Main packages were scikit‐learn 1.7.2, xgboost 3.1.1, lightgbm 4.6.0, catboost 1.2.8, pandas 2.3.3, numpy 2.3.4, and shap 0.50.0. Statistical analyses were performed using R Version 4.5.2. The threshold for statistical significance was set at *p* < 0.05.

## 3. Results

### 3.1. Baseline Characters and Grouping

**Figure 1:**
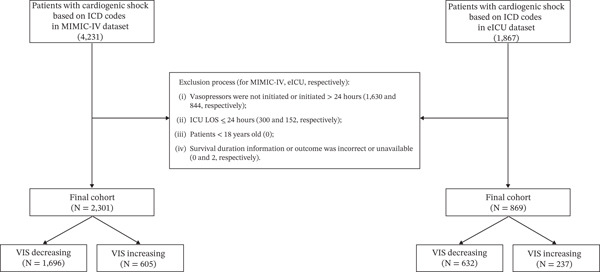
The study flowchart utilized to select the final cohort of patients.

A total of 3170 patients were included in the combined cohort, of whom 2301 were from the MIMIC‐IV database and 869 from the eICU database (Figure 1). Within each dataset, patients were further categorized according to the early VIS trajectory into a VIS‐decreasing group and a VIS‐increasing group: 1696 (73.7%) versus 605 (26.3%) patients in MIMIC‐IV and 632 (72.8%) versus 237 (27.2%) patients in eICU, respectively.

Table 1 summarizes the baseline demographic and clinical characteristics. The median age of the overall cohort was 70.0 years [60.0–78.0], with similar ages in MIMIC‐IV and eICU (71.0 [60.0–79.0] vs. 67.0 [57.0–76.0] years; *p* < 0.001). Approximately one‐third of patients were female (37.5% overall), with no significant difference between databases. Illness severity at ICU admission was high in both cohorts. Median SAPS II scores were comparable between MIMIC‐IV and eICU, whereas SOFA scores were slightly higher in eICU (10.0 [8.0–13.0] vs. 9.0 [6.0–11.0]; *p* < 0.001).

**Table 1:** Basic demographic characteristics of the original cohort.

	Overall (*N* = 3170)	MIMIC‐IV (*N* = 2301)	eICU (*N* = 869)	*p* value	Missing data (%)
Age	**70.00 [60.00, 78.00]**	**71.00 [60.00, 79.00]**	**67.00 [57.00, 76.00]**	**< 0.001**	**0.00**
Gender (female)	1190 (37.54%)	869 (37.77%)	321 (36.94%)	0.7	0.00
Race					
Asian	**77 (2.43%)**	**69 (3.00%)**	**8 (0.92%)**	**< 0.001**	**0.00**
Black	**309 (9.75%)**	**227 (9.87%)**	**82 (9.44%)**		
White	**2116 (66.75%)**	**1439 (62.54%)**	**677 (77.91%)**		
Other	**668 (21.07%)**	**566 (24.60%)**	**102 (11.74%)**		
Weight	80.00 [68.10, 95.52]	80.00 [68.00, 94.50]	80.00 [68.50, 99.00]	0.21	0.00
SAPS II	46.00 [36.00, 57.00]	46.00 [37.00, 57.00]	45.00 [34.00, 58.00]	0.22	0.00
SOFA score	**9.00 [7.00, 12.00]**	**9.00 [6.00, 11.00]**	**10.00 [8.00, 13.00]**	**< 0.001**	**0.00**
Charlson comorbidity index	**6.00 [4.00, 8.00]**	**6.00 [4.00, 8.00]**	**4.00 [2.00, 6.00]**	**< 0.001**	**0.00**
Interventions (Boolean for 1st 24 h)					
RRT (yes)	282 (8.90%)	205 (8.91%)	77 (8.86%)	1	0.00
Mechanical ventilation (yes)	**2149 (67.79%)**	**1502 (65.28%)**	**647 (74.45%)**	**< 0.001**	**0.00**
Sedative therapy (yes)	**2128 (67.13%)**	**1573 (68.36%)**	**555 (63.87%)**	**0.02**	**0.00**
Albumin (yes)	**572 (18.04%)**	**483 (20.99%)**	**89 (10.24%)**	**< 0.001**	**0.00**
Comorbidities (Boolean)					
HF (yes)	**2238 (70.60%)**	**1798 (78.14%)**	**440 (50.63%)**	**< 0.001**	**0.00**
Hypertension (yes)	**2123 (66.97%)**	**1672 (72.66%)**	**451 (51.90%)**	**< 0.001**	**0.00**
AFIB (yes)	**1762 (55.58%)**	**1250 (54.32%)**	**512 (58.92%)**	**0.02**	**0.00**
T2DM (yes)	1165 (36.75%)	844 (36.68%)	321 (36.94%)	0.93	0.00
Renal (yes)	**1177 (37.13%)**	**943 (40.98%)**	**234 (26.93%)**	**< 0.001**	**0.00**
Liver (yes)	**170 (5.36%)**	**88 (3.82%)**	**82 (9.44%)**	**< 0.001**	**0.00**
COPD (yes)	554 (17.48%)	390 (16.95%)	164 (18.87%)	0.22	0.00
CAD (yes)	**2007 (63.31%)**	**1366 (59.37%)**	**641 (73.76%)**	**< 0.001**	**0.00**
Stroke (yes)	309 (9.75%)	227 (9.87%)	82 (9.44%)	0.77	0.00
Malignancy (yes)	**135 (4.26%)**	**28 (1.22%)**	**107 (12.31%)**	**< 0.001**	**0.00**
Vital signs (1st 24 h)					
MAP	**75.00 [65.00, 87.00]**	**75.00 [65.00, 87.00]**	**73.00 [63.00, 86.00]**	**0.008**	**0.09**
Temperature	**36.56 [36.17, 36.89]**	**36.56 [36.27, 36.89]**	**36.50 [36.00, 36.90]**	**0.01**	**9.68**
Heart rate	**90.00 [77.00, 107.00]**	**89.00 [77.00, 106.00]**	**92.00 [79.00, 111.00]**	**< 0.001**	**0.00**
Laboratory tests (1st 24 h)					
WBC count	**13.20 [9.50, 18.40]**	**13.00 [9.30, 18.10]**	**13.90 [10.00, 19.20]**	**0.002**	**0.28**
Hemoglobin	**10.60 [8.80, 12.50]**	**10.40 [8.60, 12.20]**	**11.20 [9.50, 13.00]**	**< 0.001**	**0.28**
Platelet	187.00 [131.00, 251.00]	186.00 [128.00, 254.00]	189.50 [139.00, 246.00]	0.42	0.28
pH	**7.34 [7.26, 7.41]**	**7.35 [7.27, 7.41]**	**7.32 [7.23, 7.39]**	**< 0.001**	**11.29**
PO_2_	**120.00 [75.00, 254.00]**	**130.00 [78.00, 281.00]**	**102.00 [72.00, 174.00]**	**< 0.001**	**22.84**
PCO_2_	40.00 [33.50, 47.00]	40.00 [34.00, 46.00]	40.05 [33.40, 48.00]	0.32	25.02
Lactate	**2.50 [1.60, 4.30]**	**2.40 [1.60, 3.90]**	**3.20 [1.70, 6.07]**	**< 0.001**	**13.19**
Creatinine	1.58 [1.01, 2.40]	1.50 [1.00, 2.40]	1.60 [1.06, 2.40]	0.85	0.06
Outcomes (Boolean)					
ICU mortality (death)	846 (26.69%)	618 (26.86%)	228 (26.24%)	0.76	0.00
In‐hospital mortality (death)	1033 (32.59%)	739 (32.12%)	294 (33.83%)	0.38	0.00
Length of stay (days)					
ICU LOS	**4.97 [2.85, 9.10]**	**5.10 [2.92, 9.46]**	**4.62 [2.69, 8.34]**	**< 0.001**	**0.00**
Hospital LOS	**9.50 [5.06, 16.49]**	**9.93 [5.58, 17.37]**	**7.72 [3.89, 14.42]**	**< 0.001**	**0.00**

*Note:* Values are presented as mean (standard deviation) or median [Q1, Q3] for continuous variables and number (percentage) for categorical variables. Variables in bold have **p** **v** **a** **l** **u** **e** < 0.05.

Use of organ support in the first 24 h was frequent. Overall, 8.9% of patients received renal replacement therapy, and two‐thirds (67.8%) required invasive mechanical ventilation, with a significantly higher ventilation rate in eICU than in MIMIC‐IV (74.5% vs. 65.3%; *p* < 0.001). Sedative therapy was used in approximately two‐thirds of patients, and early albumin administration was more common in MIMIC‐IV than in eICU (20.99% vs. 10.24%; *p* < 0.001).

Patients had a high burden of chronic disease, dominated by cardiovascular comorbidities (heart failure, hypertension, and atrial fibrillation), with other conditions such as diabetes, chronic kidney disease, COPD, and coronary artery disease also common. In the first 24 h after ICU admission, vital signs indicated moderate hemodynamic and respiratory instability, with low‐normal MAP and generally elevated heart rate. Initial laboratory tests showed systemic inflammation and metabolic/respiratory disturbances on arterial blood gas analysis, including increased lactate, consistent with circulatory compromise. Overall, these findings reflect a severely ill population with substantial comorbidity and early organ dysfunction.

Clinical outcomes were poor in both datasets. Overall, ICU mortality was 26.7%, and in‐hospital mortality was 32.6%, with no significant differences between MIMIC‐IV and eICU. However, length of stay differed: ICU stay was shorter in eICU (median 4.62 [2.69–8.34] vs. 5.10 [2.92–9.46] days; *p* < 0.001), and hospital LOS was also shorter (7.72 [3.89–14.42] vs. 9.93 [5.58–17.37] days; *p* < 0.001). Tables S1 and S2 provide detailed baseline characteristics of the MIMIC‐IV and eICU cohorts stratified by VRR.

Taken together, these findings indicate that although MIMIC‐IV and eICU populations differed in demographic profile, comorbidity burden, and some early physiological and laboratory parameters, severity scores and mortality rates were broadly comparable. This supported the use of MIMIC‐IV as the derivation cohort and eICU as an independent external validation cohort for assessing the prognostic value of VRR and related prediction models.

### 3.2. Clinical Outcomes and Sensitivity Analyses

In both cohorts, patients in the VIS‐increasing group experienced substantially worse outcomes than those in the VIS‐decreasing group. In the MIMIC‐IV cohort, the Kaplan–Meier curves for in‐hospital survival showed a marked separation between the two VIS trajectories, with significantly higher in‐hospital mortality in the VIS‐increasing group (log‐rank *p* < 0.001; Figure 2a). Consistent with these unadjusted findings, the VIS‐increasing group was associated with a higher risk of in‐hospital, ICU, and 28‐day mortality across all regression models (Table 2, Figures S1A and S2). In the log‐rank model (Model 1), VIS‐increasing status was associated with an 82% higher in‐hospital mortality risk (HR 1.82, 95% CI 1.54–2.14; *p* < 0.001), a 67% higher ICU mortality risk (HR 1.67, 95% CI 1.41–1.99; *p* < 0.001), and more than a two‐fold increase in 28‐day mortality (HR 2.12, 95% CI 1.80–2.50; *p* < 0.001). These associations remained robust after adjustment for all candidate covariates in Model 2 and after adjustment for covariates selected by univariable analyses in Model 3.

Figure 2Unadjusted Kaplan–Meier curve for primary outcome. (a) Kaplan–Meier curve of in‐hospital mortality for the MIMIC‐IV cohort. (b) Kaplan–Meier curve of in‐hospital mortality for the eICU cohort.

**Figure:**
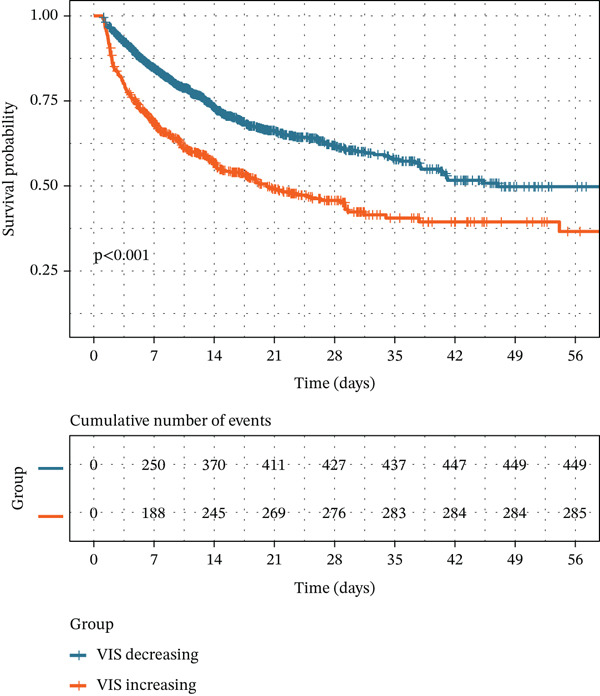
(a)

**Figure:**
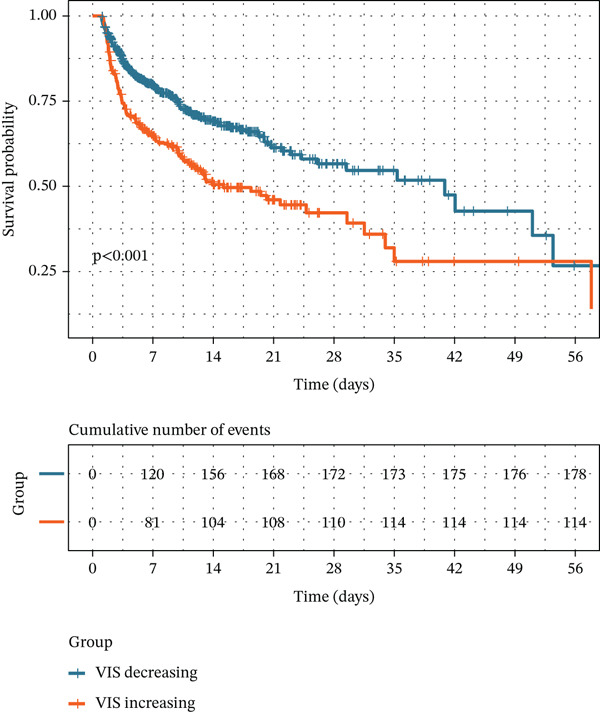
(b)

**Table 2:** Primary and secondary outcome analyses with different models for the MIMIC‐IV cohort.

	Model		In‐hospital mortality		ICU mortality		28‐day mortality	
		Group	*p* value	Result	*p* value	Result	*p* value	Result
MIMIC‐IV cohort	Model 1	VIS‐decreasing						
		VIS‐increasing	**< 0.001**	**1.82 (1.54, 2.14)**	**< 0.001**	**1.67 (1.41, 1.99)**	**< 0.001**	**2.12 (1.80, 2.50)**
	Model 2	VIS‐decreasing						
		VIS‐increasing	**< 0.001**	**1.79 (1.54, 2.09)**	**< 0.001**	**1.69 (1.43, 2.00)**	**< 0.001**	**2 (1.73, 2.32)**
	Model 3	VIS‐decreasing						
		VIS‐increasing	**< 0.001**	**1.79 (1.54, 2.08)**	**< 0.001**	**1.68 (1.42, 1.98)**	**< 0.001**	**2.02 (1.75, 2.34)**
	Model 4	VIS‐decreasing						
		VIS‐increasing	**< 0.001**	**1.79 (1.54, 2.08)**	**< 0.001**	**1.68 (1.42, 1.98)**	**< 0.001**	**2.03 (1.76, 2.34)**
eICU cohort	Model 1	VIS‐decreasing						
		VIS‐increasing	**< 0.001**	**1.75 (1.35, 2.26)**	**< 0.001**	**1.71 (1.29, 2.28)**		
	Model 2	VIS‐decreasing						
		VIS‐increasing	**< 0.001**	**1.79 (1.39, 2.30)**	**< 0.001**	**1.92 (1.44, 2.55)**		
	Model 3	VIS‐decreasing						
		VIS‐increasing	**< 0.001**	**1.87 (1.46, 2.39)**	**< 0.001**	**1.95 (1.47, 2.57)**		
	Model 4	VIS‐decreasing						
		VIS‐increasing	**< 0.001**	**1.75 (1.37, 2.24)**	**< 0.001**	**1.82 (1.38, 2.41)**		

*Note:* Statistical analyses of different models with *p* value < 0.05 were displayed in bold. Model 1: log‐rank model (HR [95% CI]). Model 2: Cox model adjusted with all covariates (HR [95% CI]). Model 3: Cox model adjusted with covariates selected by univariable analyses (HR [95% CI]). Model 4: Cox model adjusted with covariates selected by random forest algorithm (HR [95% CI]).

Abbreviations: CI, confidence interval; HR, hazard ratio.

To further reduce model complexity and potential overfitting, we constructed Model 4 using covariates identified by the Boruta random forest feature selection algorithm (Figure S3A). The variables retained by Boruta mainly reflected global illness severity and early organ dysfunction, including SAPS II, SOFA score, lactate, creatinine, age, Charlson comorbidity index, and key treatment/intervention variables. After adjustment for these Boruta‐selected covariates, VIS‐increasing status remained independently associated with higher risks of in‐hospital mortality (HR 1.79, 95% CI 1.54–2.08; *p* < 0.001), ICU mortality (HR 1.68, 95% CI 1.42–1.98; *p* < 0.001), and 28‐day mortality (HR 2.03, 95% CI 1.76–2.34; *p* < 0.001) in the MIMIC‐IV cohort.

In the external eICU cohort, the association between an increasing VIS trajectory and adverse outcomes was broadly consistent with that observed in MIMIC‐IV. Patients in the VIS‐increasing group had significantly higher risks of in‐hospital and ICU mortality across all models (Table 2, Figure 2b, and Figure S1B). In the log‐rank model (Model 1), VIS‐increasing status was associated with a 75% higher in‐hospital mortality risk (HR 1.75, 95% CI 1.35–2.26; *p* < 0.001) and a 71% higher ICU mortality risk (HR 1.71, 95% CI 1.29–2.28; *p* < 0.001). After adjustment with Boruta‐selected covariates in Model 4 (Figure S3B), VIS‐increasing status remained independently associated with in‐hospital mortality (HR 1.75, 95% CI 1.37–2.24; *p* < 0.001) and ICU mortality (HR 1.82, 95% CI 1.38–2.41; *p* < 0.001) in the eICU cohort.

Taken together, these results demonstrate that an early increasing VRR is a strong and consistent predictor of adverse outcomes, including in‐hospital, ICU, and 28‐day mortality (Tables S3–S22), and that this association remains stable after rigorous adjustment using different modeling strategies and data‐driven covariate selection in both the derivation (MIMIC‐IV) and external validation (eICU) cohorts.

### 3.3. Subgroup Analysis

Subgroup analyses consistently showed that an increasing VIS trajectory was associated with higher mortality across clinically relevant strata (Figure 3). For in‐hospital mortality, the elevated risk in the VIS‐increasing group persisted in both the MIMIC‐IV (Figure 3a) and eICU cohorts (Figure 3b), with HRs > 1 in most examined subgroups, which suggests that the detrimental impact of an increasing VIS trajectory was stable across these baseline characteristics.

Figure 3Forest plot of subgroup analysis. (a) Forest plot of subgroup analysis of in‐hospital mortality for the MIMIC‐IV cohort. (b) Forest plot of subgroup analysis of in‐hospital mortality for the eICU cohort.

**Figure:**
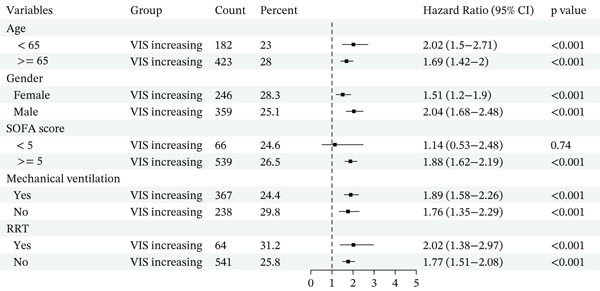
(a)

**Figure:**
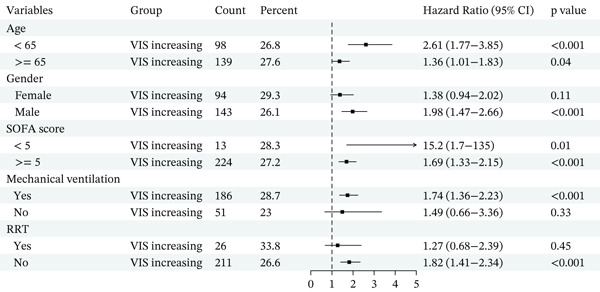
(b)

Similar patterns were observed when ICU mortality was used as the endpoint. In both MIMIC‐IV (Figure S4A) and eICU (Figure S4B), VIS‐increasing status remained significantly associated with a higher risk of ICU death in nearly all subgroups. In the MIMIC‐IV cohort, subgroup analyses for 28‐day mortality (Figure S5) were consistent with the primary analyses. Across all predefined strata of age, sex, illness severity, mechanical ventilation, and renal replacement therapy, patients in the VIS‐increasing group had markedly higher 28‐day mortality.

Overall, these subgroup analyses indicate that the adverse prognostic significance of an early increasing VIS trajectory is robust and broadly generalizable, rather than being confined to any specific patient subgroup.

### 3.4. Interpretable Machine Learning Models

In the internal validation set, CatBoost, LightGBM, and XGBoost yielded the highest AUCs (Figure 4). In the external eICU cohort, overall AUCs were slightly attenuated, but the ranking of algorithms remained largely consistent, indicating acceptable generalizability. Pairwise AUC difference heatmaps demonstrated that gradient boosting models achieved statistically higher AUCs than linear models and Gaussian Naive Bayes (Figure 4). Because all algorithms were trained on the same feature set and data partitions, these performance gains are likely attributable to the ability of nonlinear models to capture complex relationships and interactions involving VRR. This suggests that incorporating VRR into conventional critical‐care variables provides additional prognostic information about the quality and trajectory of hemodynamic resuscitation.

Figure 4Comparison of model performance for predicting mortality in cardiogenic shock patients. (a) ROC curves and AUCs of all models for the MIMIC‐IV cohort. (b) ROC curves and AUCs of all models for the eICU cohort. (c) Heatmap showing pairwise AUC differences between models for the MIMIC‐IV cohort. (d) Heatmap showing pairwise AUC differences between models for the eICU cohort.

**Figure:**
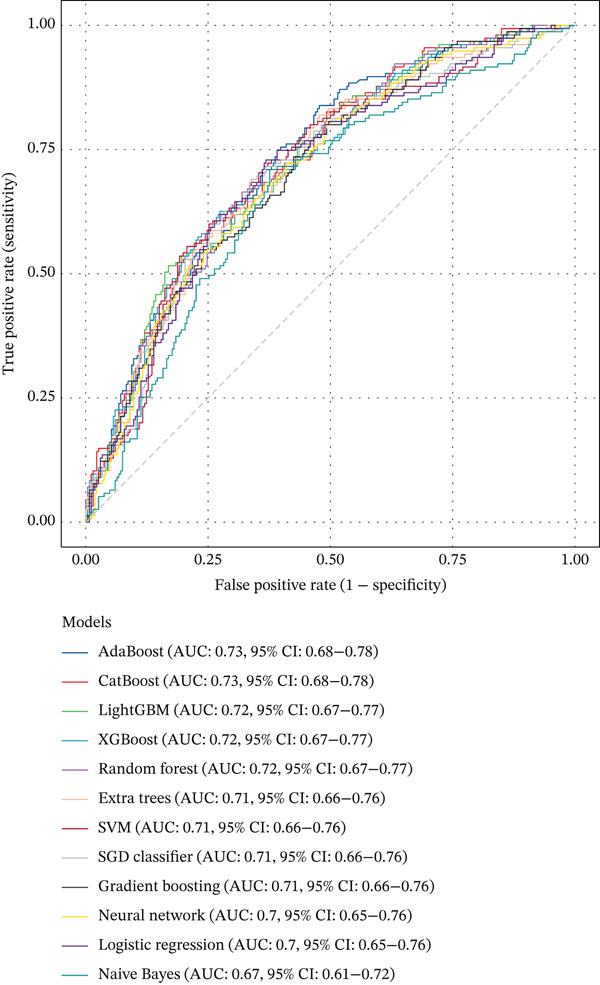
(a)

**Figure:**
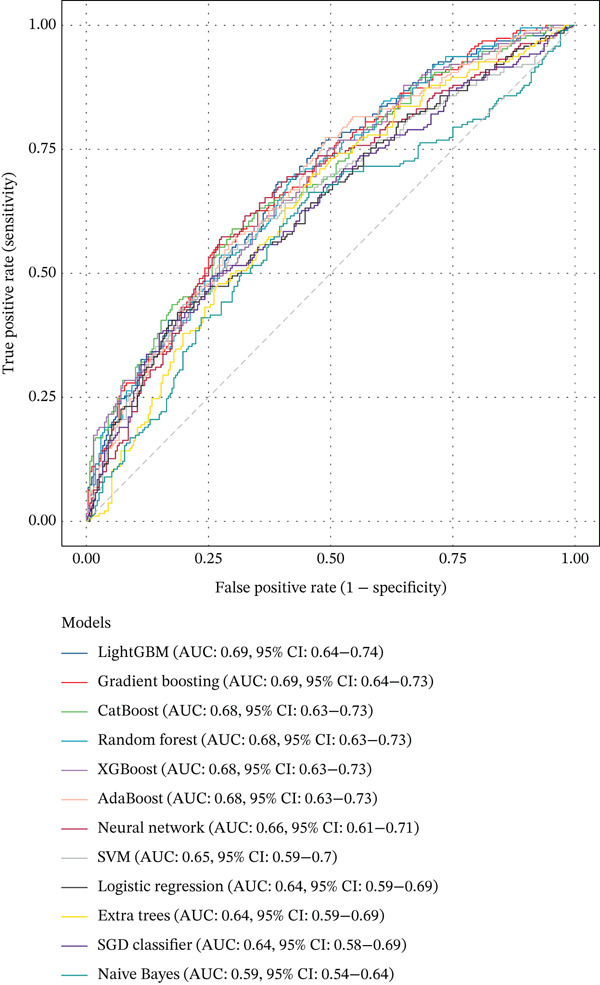
(b)

**Figure:**
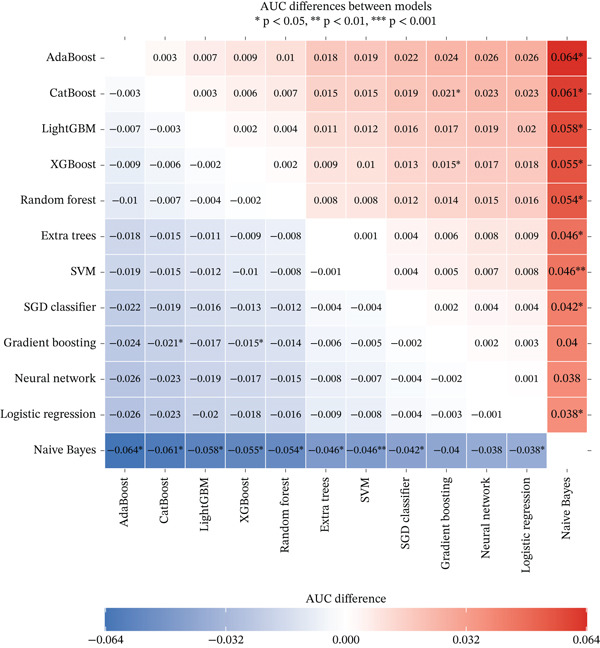
(c)

**Figure:**
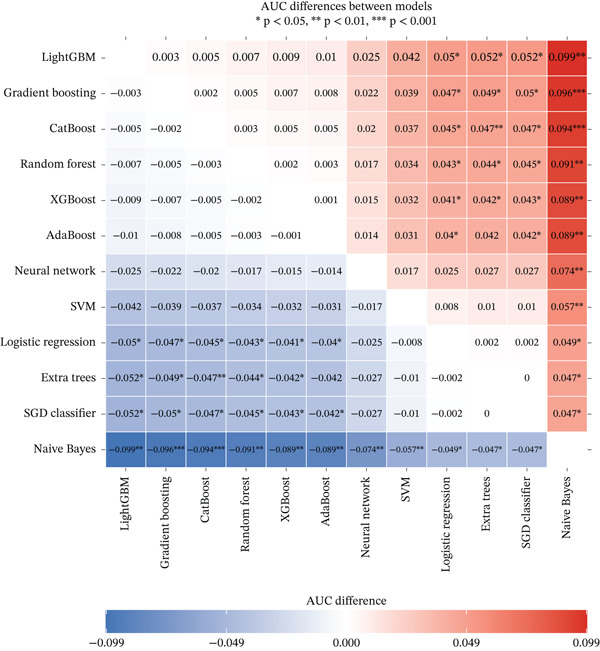
(d)

For all tree‐based models, we applied SHAP to quantify the contribution of each feature to the prediction of in‐hospital mortality. In the CatBoost model, VRR emerged as the most influential feature in the internal cohort, with the largest mean absolute SHAP value among all predictors (Figure 5). VRR ranked above age, lactate, creatinine, hemoglobin, weight, and vital signs. In the external eICU cohort, VRR again occupied the top position or was tied with lactate and age, confirming its dominant contribution to the prediction of in‐hospital mortality. Similar rankings were observed across other tree‐based algorithms (Figures S6 and S7), indicating that the prognostic signal carried by VRR is robust and model‐independent.

Figure 5SHAP (SHapley Additive exPlanations) summary plots showing the contribution of the top features to the CatBoost model predictions. (a) SHAP summary plots of the CatBoost model for the MIMIC‐IV cohort. (b) SHAP summary plots of the CatBoost model for the eICU cohort.

**Figure:**
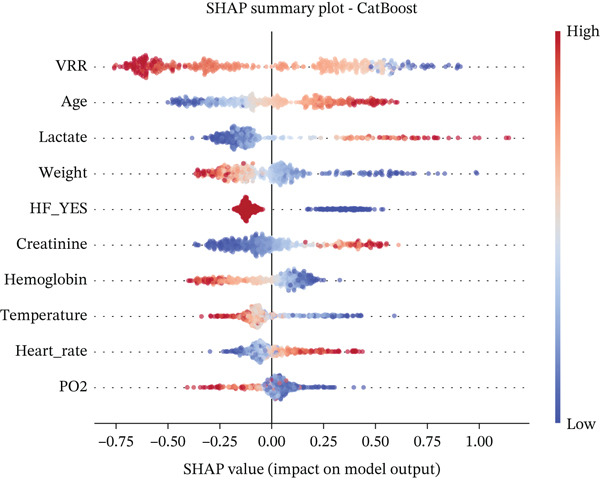
(a)

**Figure:**
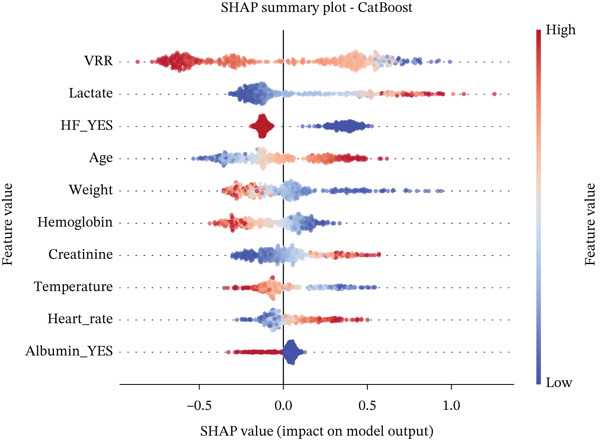
(b)

SHAP summary plots further revealed a clear and clinically coherent pattern: higher VRR values were predominantly associated with negative or near‐zero SHAP values, indicating a protective effect that reduced the predicted probability of death. In contrast, lower VRR values clustered on the positive SHAP, consistently pushing the model toward a high‐risk classification.

## 4. Discussion

Our retrospective study revealed that VIS reduction strongly correlates with better outcomes. Using VRR to track VIS dynamics, we found that patients in the decreased VIS group experienced significantly lower mortality rates compared to those with increased VIS. This establishes VRR as a valuable dynamic monitoring tool for risk stratification and clinical guidance.

Previous studies have demonstrated that the combined assessment of VIS and lactate levels serves as a prognostic predictor for patients with postcardiac surgery CS requiring venoarterial ECMO support [15]. The vasoactive–ventilation–renal (VVR) score has not only proven valuable in prognostic assessment following pediatric cardiac surgery [16] but has also been validated as an effective early prediction tool for adult patients undergoing mitral valve surgery [17]. However, most of these prior works have focused on static VIS values at fixed time points (e.g., a maximum or cumulative VIS within the first 24 h) [18, 19]. Such approaches provide important information about the early intensity of vasoactive support but do not fully capture how vasoactive requirements evolve over time. In contrast, our study centers on VRR as a dynamic index that reflects the trajectory of vasoactive support rather than a single static value. From a physiological standpoint, VRR summarizes whether a patient can be successfully weaned from high‐dose vasoactive therapy (favorable VRR) or instead requires escalating doses to maintain hemodynamic stability (unfavorable VRR). This temporal dimension complements the information provided by lactate clearance, which primarily reflects global tissue perfusion and metabolic recovery, and by composite indices such as the VVR score, which integrates cardiovascular, respiratory, and renal components into a single metric. This study emphasizes the critical importance of real‐time monitoring and adjustment of vasoactive medications in improving patient outcomes. Through analysis of dynamic trends in VRR scores, clinicians can promptly evaluate disease progression in patients with CS in the ICU. This assessment approach provides ICU healthcare teams with a rapid and reliable tool for identifying high‐risk patients and implementing timely interventions, thereby reducing mortality risk. In our machine learning models, VRR was evaluated alongside lactate levels, other laboratory markers, and conventional severity scores and remained an important predictor of mortality even when entered together with these variables. We therefore propose that VRR should be interpreted in conjunction with these established dynamic indices as part of a multimodal monitoring strategy for patients with CS.

CS represents the most severe complication of cardiovascular disease, characterized by high mortality. Its progression affects the entire circulatory system, leading to complications such as tissue injury, inflammation, and hypoxia. It triggers compensatory mechanisms, including endogenous sympathetic stimulation, peripheral vasoconstriction, and increased heart rate and myocardial contractility. Over time, continuous increases in cardiac afterload and a mismatch between myocardial oxygen supply and demand can lead to a decompensated stage, reducing coronary perfusion [20]. Thus, a hemodynamic support strategy utilizing vasoactive medications is vital to enhance tissue perfusion and prevent worsening myocardial ischemia, which is a consensus in clinical practice. While these endogenous compounds produce direct cardiac effects, peripheral vasoconstriction increases systemic vascular resistance and MAP. However, these compensatory responses are accompanied by maladaptive increases in cardiac afterload, myocardial oxygen requirements, and filling pressures, ultimately reducing coronary perfusion pressure [21]. Therefore, prompt hemodynamic support is crucial to restore cellular metabolism and prevent further systemic and myocardial ischemia, thus confirming the necessity of vasoactive agent therapy for patients with CS.

Vasopressors or inotropes are frequently administered to patients with CS, with approximately 90% of these patients receiving vasoactive medications [20]. However, different vasopressors yield varying outcomes. Norepinephrine, epinephrine, phenylephrine, and dopamine are potent vasopressors with similar effects in increasing MAP [22]. Levosimendan provides peripheral vasodilatory effects and enhances myocardial contractility through vascular smooth muscle potassium channel binding and cardiac myofilament calcium sensitization [23]. Orprinone hydrochloride, a third‐generation phosphodiesterase III inhibitor, has been proven safe and effective in treating heart failure [24]. Methylene blue, initially used for methemoglobinemia treatment, has gained interest for refractory cardiac shock treatment by inhibiting nitric oxide‐mediated cGMP production, leading to increased smooth muscle vasoconstriction [25].

Additionally, a randomized controlled trial demonstrated that epinephrine administration is more likely to lead to refractory CS than norepinephrine in patients with CS‐complicating AMI [26]. Mathew et al. enrolled 192 CS patients who were randomly assigned to receive either dobutamine or milrinone, finding no significant difference between the drugs in terms of in‐hospital mortality, resuscitated cardiac arrest, cardiac transplantation or mechanical circulatory support, nonfatal myocardial infarction, stroke, or renal replacement therapy [27]. Conversely, a recent study by Rodenas‐Alesina et al. showed that milrinone benefits CS patients more than dobutamine [28]. There is an increasing discussion regarding a tailored vasopressor treatment strategy for individual patients, emphasizing the rational use of vasoactive agents [29]. These agents, with their narrow therapeutic spectrum, could lead to potentially lethal complications. Therefore, precise therapeutic targets, close monitoring, and titration to the minimum effective dose are essential. Vasoactive agents should be weaned as promptly as possible. Administering these agents in CS requires an individualized approach. Conducting randomized controlled trials in CS populations has been historically challenging, leading to societal recommendations based on small trials, meta‐analyses, and consensus opinions, resulting in equipoise concerning the recommended first‐line vasoactive agents for CS treatment. Therefore, there is no consensus on the benefit of vasoactive agents in improving the short‐term prognosis of CS patients.

VIS objectively quantifies the degree of hemodynamic support. Belletti et al. [10] updated the VIS in 2020, adding enoximone, levosimendan, olprinone, methylene blue, phenylephrine, terlipressin, and angiotensin II, allowing a more comprehensive quantitative assessment of vasoactive agent dosages. In clinical practice, various vasoactive substances are used for CS patients, and VIS is a valuable hemodynamic scoring system. Vasoactive agents can indicate the severity of CS. Numerous studies have shown a significant correlation between higher early VIS and increased mortality in patients with CS and in cardiac surgery patients [30]. A retrospective study using the MIMIC‐IV database demonstrated a higher risk of ICU and in‐hospital mortality in patients with septic shock associated with vasopressor/inotropic agents [8].

Previous research on VIS was limited to single measurements assessing its association with mortality. Our study focused on variations in VIS, specifically the reduction in vasoactive agent infusion rates and their impact on mortality in patients with CS. We utilized high‐resolution data for a large sample size and confirmed the association between early reduction in vasoactive agent dosages and improved clinical outcomes. This study demonstrates that a more significant decrease in VIS is associated with lower mortality, indicating that dynamic changes in VIS within 48 h of ICU admission can be used to assess patient prognosis.

From a clinical perspective, our findings suggest that VRR may serve as an adjunctive dynamic marker that can be integrated into risk‐prediction models or clinical decision support tools. Such tools could continuously update estimated mortality risk as vasoactive requirements change over time and flag patients with persistently low or negative VRR—indicating failure to reduce vasoactive support despite ongoing treatment—for closer reassessment. In this context, a low or worsening VRR might prompt a more systematic evaluation of hemodynamics, reconsideration of the underlying etiology of shock, optimization of fluid status and afterload reduction, or early discussion of mechanical circulatory support, rather than directly dictating specific therapeutic actions. To further explore the potential bedside utility of VRR, we developed machine learning models for in‐hospital mortality that incorporated VRR alongside conventional static and dynamic variables. These models were trained and internally validated in MIMIC‐IV and externally validated in eICU, and VRR consistently emerged as an important predictor, suggesting that it provides additional prognostic information beyond standard clinical measures. Nonetheless, our work remains exploratory and hypothesis‐generating. Prospective, preferably multicenter interventional studies are required to determine whether VRR‐guided strategies (e.g., predefined VRR thresholds to trigger intensified monitoring or escalation of support) are feasible, safe, and effective in improving outcomes and to evaluate the real‐world practicality of integrating VRR‐based decision support into ICU workflows.

There are several limitations in our study that need to be addressed. Firstly, we used the MIMIC‐IV and eICU databases to evaluate the relationship between vasopressor use and in‐hospital mortality, ICU mortality, and 28‐day mortality (the latter assessed only in the MIMIC‐IV database). In addition, both are United States–based critical care databases. Therefore, caution is needed when extrapolating our findings to other healthcare systems, countries, or resource‐limited settings. Secondly, CS was identified using ICD codes, and we additionally required exposure to vasoactive and/or inotropic agents consistent with the management of CS. Although the combination of ICD codes with vasoactive use likely provides the most accurate and standardized case definition feasible in large retrospective databases, some degree of diagnostic misclassification is still possible and may have influenced our findings. Nevertheless, the consistent association between VRR and mortality observed in both the single‐center MIMIC‐IV cohort and the multicenter eICU cohort supports the robustness and external validity of our results despite potential imperfections in any individual coding system. Thirdly, not all vasoactive or inotropic agents included in the 2020 VIS update were consistently available in the MIMIC‐IV and eICU databases. In particular, drugs such as levosimendan and olprinone were either not captured or used only rarely, which may lead to a slight underestimation of total vasoactive burden in some patients. Patient responses to different vasopressor agents are neither uniform nor predictable, which could influence the results [31]. However, the core agents that form the backbone of contemporary CS management in adult ICUs—dopamine, dobutamine, epinephrine, norepinephrine, phenylephrine, and milrinone—were well represented in both databases. Thus, our VIS and VRR calculations already incorporate the major contributors to vasoactive support in this clinical context. By applying the VIS 2020, we standardized the quantification of vasoactive “intensity” across heterogeneous drug regimens, allowing different combinations and dosing strategies to be translated into a comparable numeric index. Importantly, despite differences in drug availability and prescribing patterns between the single‐center MIMIC‐IV cohort and the multicenter eICU cohort, we observed a consistent association between VRR and mortality in both databases. This concordance suggests that, even with incomplete coverage of a few infrequently used agents, VRR remains a robust and generalizable dynamic marker that can objectively summarize the overall intensity of vasoactive and inotropic therapy across diverse clinical environments. In addition, substantial patient heterogeneity remains an important limitation. Owing to the inherent constraints of the MIMIC‐IV and eICU databases, we were not able to determine the precise primary etiology of CS for every individual patient, nor could we comprehensively capture all forms of mechanical circulatory support (e.g., detailed information on ECMO and Impella is not reliably available, particularly in eICU). We attempted to mitigate this heterogeneity by adjusting for a broad set of baseline comorbidities (such as chronic kidney disease, chronic liver disease, and heart failure), disease severity indicators (including SOFA score and other available physiologic and laboratory variables), and major concurrent interventions (mechanical ventilation and renal replacement therapy), and by performing subgroup analyses across clinically relevant strata. The inverse association between VRR and mortality was generally consistent in both direction and magnitude across these subgroups and in both databases, which supports the robustness of our findings. Nevertheless, residual confounding related to unmeasured or imperfectly measured factors cannot be excluded and should be considered.

## 5. Conclusion

In our retrospective observational study, VRR emerged as a key dynamic hemodynamic indicator associated with patient outcomes in VIS data. Among CS patients receiving vasopressors for more than 24 h, higher VRR was associated with lower in‐hospital, ICU, and 28‐day mortality. These findings underscore the importance of monitoring and managing VRR as a dynamic hemodynamic index and highlight the need for more refined vasopressor management that takes into account previously overlooked hemodynamic dynamics in the ICU.

**Nomenclature**
CS: cardiogenic shock
AMI: acute myocardial infarction
CO: cardiac output
BP: blood pressure
VIS: vasoactive–inotropic score
ECMO: extracorporeal membrane oxygenation
ICD: International Classification of Diseases
VRR: VIS reduction rate
ICU: intensive care unit
SAPS II: Simplified Acute Physiology Score II
SOFA: Sequential Organ Failure Assessment
HF: heart failure
AFIB: atrial fibrillation
T2DM: Type 2 diabetes
COPD: chronic obstructive pulmonary disease
CAD: coronary artery disease
MAP: mean arterial pressure
WBC: white blood cell
pH: potential of hydrogen
PO_2_
: partial pressure of oxygen
PCO_2_
: partial pressure of carbon dioxide
SVM: support vector machine
ROC: receiver operator characteristic
AUC: area under the ROC curve
SHAP: SHapley Additive exPlanations
HRs: hazard ratios
CIs: confidence intervals

## Author Contributions

L‐X.Z. and Q‐Q.M. designed this study. The SQL and R code were executed by Y‐L.N., T‐X.G., and Q‐Q.M. The machine learning Python code was implemented by Y‐L.N. Data validation and manuscript drafting and revision were conducted by Y‐L.N., T‐X.G., X‐L.N., Q‐Q.M., Y‐N.Z., and T‐Y.P. L‐X.Z. contributed to the interpretation of the data. L‐X.Z., H‐T.G., and H.L. served as senior supervisors of the project and provided critical revisions to the manuscript. Y‐L.N. and T‐X.G. contributed equally to this work and shared the first authorship.

## Funding

This study was funded by the National Natural Science Foundation of China (10.13039/501100001809) (Nos. 72404064 and 82304939), Shenzhen Science and Technology Innovation Program (RCBS20231211090824039), the High Quality Healthcare Team Project of Shenzhen Bao′an Chinese Medicine Hospital, the Sanming Project of Shenzhen Bao′an Chinese Medicine Hospital, the National Project for the Development of Key Specialties in Chinese Medicine (900), and the 2024 High‐Quality Development Research Project of Shenzhen Bao′an Public Hospital (BAGZL2024117).

## Disclosure

All authors thoroughly reviewed and approved the final version of the manuscript and agreed to assume full responsibility for the research presented.

## Ethics Statement

Y‐L.N. possesses complete access to the MIMIC‐IV and eICU databases, which were established with approval from both the Massachusetts Institute of Technology (located in Cambridge, MA) and Beth Israel Deaconess Medical Center (situated in Boston, MA). Consent was secured during the original data collection process. Consequently, the requirement for ethical approval and informed consent was exempted for this manuscript.

## Conflicts of Interest

The authors declare no conflicts of interest.

## Supporting information


**Supporting Information** Additional supporting information can be found online in the Supporting Information section. Figure S1 Unadjusted Kaplan–Meier curve for secondary outcome. (A) Kaplan–Meier curve of ICU mortality for the MIMIC‐IV cohort. (B) Kaplan–Meier curve of ICU mortality for the eICU cohort. Figure S2: Unadjusted Kaplan–Meier curve for 28‐day outcome. Figure S3: Feature selection results using the Boruta algorithm. (A) Relative importance of candidate predictors for in‐hospital mortality for the MIMIC‐IV cohort. (B) Relative importance of candidate predictors for in‐hospital mortality for the eICU cohort. Figure S4: Forest plot of subgroup analysis. (A) Forest plot of subgroup analysis of ICU mortality for the MIMIC‐IV cohort. (B) Forest plot of subgroup analysis of ICU mortality for the eICU cohort. Figure S5: Forest plot of subgroup analysis of 28‐day mortality for the MIMIC‐IV cohort. Figure S6: SHAP (SHapley Additive exPlanations) summary plots showing the contribution of the top features to the LightGBM model predictions. (A) SHAP summary plots of the LightGBM model for the MIMIC‐IV cohort. (B) SHAP summary plots of the LightGBM model for the eICU cohort. Figure S7: SHAP (SHapley Additive exPlanations) summary plots showing the contribution of the top features to the XGBoost model predictions. (A) SHAP summary plots of the XGBoost model for the MIMIC‐IV cohort. (B) SHAP summary plots of the XGBoost model for the eICU cohort. Table S1: Basic demographic characteristics of the MIMIC‐IV cohort. Table S2: Basic demographic characteristics of the eICU cohort. Table S3: Unadjusted log‐rank test for in‐hospital mortality of the MIMIC‐IV cohort. Table S4: Unadjusted log‐rank test for ICU mortality of the MIMIC‐IV cohort. Table S5: Unadjusted log‐rank test for 28‐day mortality of the MIMIC‐IV cohort. Table S6: Unadjusted log‐rank test for in‐hospital mortality of the eICU cohort. Table S7: Unadjusted log‐rank test for ICU mortality of the eICU cohort. Table S8: Multivariate Cox model adjusted with all covariates for in‐hospital mortality of the MIMIC‐IV cohort. Table S9: Multivariate Cox model adjusted with covariates selected by univariable analyses for in‐hospital mortality of the MIMIC‐IV cohort. Table S10: Multivariate Cox model adjusted with covariates selected by the Boruta algorithm for in‐hospital mortality of the MIMIC‐IV cohort. Table S11: Multivariate Cox model adjusted with all covariates for ICU mortality of the MIMIC‐IV cohort. Table S12: Multivariate Cox model adjusted with covariates selected by univariable analyses for ICU mortality of the MIMIC‐IV cohort. Table S13: Multivariate Cox model adjusted with covariates selected by the Boruta algorithm for ICU mortality of the MIMIC‐IV cohort. Table S14: Multivariate Cox model adjusted with all covariates for 28‐day mortality of the MIMIC‐IV cohort. Table S15: Multivariate Cox model adjusted with covariates selected by univariable analyses for 28‐day mortality of the MIMIC‐IV cohort. Table S16: Multivariate Cox model adjusted with covariates selected by the Boruta algorithm for 28‐day mortality of the MIMIC‐IV cohort. Table S17: Multivariate Cox model adjusted with all covariates for in‐hospital mortality of the eICU cohort. Table S18: Multivariate Cox model adjusted with covariates selected by univariable analyses for in‐hospital mortality of the eICU cohort. Table S19: Multivariate Cox model adjusted with covariates selected by the Boruta algorithm for in‐hospital mortality of the eICU cohort. Table S20: Multivariate Cox model adjusted with all covariates for ICU mortality of the eICU cohort. Table S21: Multivariate Cox model adjusted with covariates selected by univariable analyses for ICU mortality of the eICU cohort. Table S22: Multivariate Cox model adjusted with covariates selected by the Boruta algorithm for ICU mortality of the eICU cohort. Table S23: Machine learning model performance in the MIMIC‐IV cohort. Table S24: Machine learning model performance in the eICU cohort.

## Data Availability

The MIMIC‐IV database is openly accessible on PhysioNet (accessible at https://www.physionet.org/). Additionally, concept codes can be found in the MIMIC Code Repository (available at https://github.com/MIT-LCP/mimic-code/).
