# Supplementary material for: Vasoactive–Inotropic Score Reduction Rate Is Highly Associated With Prognosis for Critically Ill Patients With Cardiogenic Shock: Insights From the Real‐World Dynamic Data
Source: Cardiovasc Ther. 2026 Mar 6;2026:8989505. doi: 10.1155/cdr/8989505 (PMC12966616; doi:10.1155/cdr/8989505)
Supplement: Supplementary file 1 — Supporting Information Additional supporting information can be found online in the Supporting Information section. Figure S1 Unadjusted Kaplan–Meier curve for secondary outcome. (A) Kaplan–Meier curve of ICU mortality for the MIMIC‐IV cohort. (B) Kaplan–Meier curve of ICU mortality for the eICU cohort. Figure S2: Unadjusted Kaplan–Meier curve for 28‐day outcome. Figure S3: Feature selection results using the Boruta algorithm. (A) Relative importance of candidate predictors for in‐hospital mortality for the MIMIC‐IV cohort. (B) Relative importance of candidate predictors for in‐hospital mortality for the eICU cohort. Figure S4: Forest plot of subgroup analysis. (A) Forest plot of subgroup analysis of ICU mortality for the MIMIC‐IV cohort. (B) Forest plot of subgroup analysis of ICU mortality for the eICU cohort. Figure S5: Forest plot of subgroup analysis of 28‐day mortality for the MIMIC‐IV cohort. Figure S6: SHAP (SHapley Additive exPlanations) summary plots showing the contribution of the top features to the LightGBM model predictions. (A) SHAP summary plots of the LightGBM model for the MIMIC‐IV cohort. (B) SHAP summary plots of the LightGBM model for the eICU cohort. Figure S7: SHAP (SHapley Additive exPlanations) summary plots showing the contribution of the top features to the XGBoost model predictions. (A) SHAP summary plots of the XGBoost model for the MIMIC‐IV cohort. (B) SHAP summary plots of the XGBoost model for the eICU cohort. Table S1: Basic demographic characteristics of the MIMIC‐IV cohort. Table S2: Basic demographic characteristics of the eICU cohort. Table S3: Unadjusted log‐rank test for in‐hospital mortality of the MIMIC‐IV cohort. Table S4: Unadjusted log‐rank test for ICU mortality of the MIMIC‐IV cohort. Table S5: Unadjusted log‐rank test for 28‐day mortality of the MIMIC‐IV cohort. Table S6: Unadjusted log‐rank test for in‐hospital mortality of the eICU cohort. Table S7: Unadjusted log‐rank test for ICU mortality of the eICU cohort. Table [file CDR-2026-8989505-s001.zip › Supplementary Tables.docx]

Supplementary Table 1. Basic demographic characteristics of the MIMIC-IV cohort

|  | **Overall (N=2301)** | **VIS decreasing (N=1696)** | **VIS increasing (N=605)** | **p-value** | **Missing data (%)** |
| --- | --- | --- | --- | --- | --- |
| Age | **71.00 [60.00, 79.00]** | **70.00 [60.00, 78.00]** | **71.00 [62.00, 80.00]** | **0.005** | **0.00** |
| Gender (Female) | 869 (37.77%) | 623 (36.73%) | 246 (40.66%) | 0.1 | 0.00 |
| Race |  |  |  |  |  |
| Asian | 69 (3.00%) | 57 (3.36%) | 12 (1.98%) | 0.4 | 0.00 |
| Black | 227 (9.87%) | 166 (9.79%) | 61 (10.08%) |  |  |
| White | 1439 (62.54%) | 1058 (62.38%) | 381 (62.98%) |  |  |
| Other | 566 (24.60%) | 415 (24.47%) | 151 (24.96%) |  |  |
| Weight | 80.00 [68.00, 94.50] | 80.04 [67.92, 94.81] | 79.92 [68.50, 93.90] | 0.93 | 0.00 |
| SAPS II | **46.00 [37.00, 57.00]** | **45.00 [37.00, 55.25]** | **47.00 [38.00, 59.00]** | **0.003** | **0.00** |
| SOFA score | 9.00 [6.00, 11.00] | 9.00 [6.00, 11.00] | 9.00 [6.00, 11.00] | 0.11 | 0.00 |
| Charlson comorbidity index | **6.00 [4.00, 8.00]** | **6.00 [4.00, 8.00]** | **7.00 [5.00, 9.00]** | **<0.001** | **0.00** |
| **Interventions (boolean for 1st 24 h)** |  |  |  |  |  |
| RRT (YES) | 205 (8.91%) | 141 (8.31%) | 64 (10.58%) | 0.11 | 0.00 |
| Mechanical ventilation (YES) | **1502 (65.28%)** | **1135 (66.92%)** | **367 (60.66%)** | **0.006** | **0.00** |
| Sedative therapy (YES) | **1573 (68.36%)** | **1182 (69.69%)** | **391 (64.63%)** | **0.02** | **0.00** |
| Albumin (YES) | **483 (20.99%)** | **381 (22.46%)** | **102 (16.86%)** | **0.004** | **0.00** |
| **Comorbidities (boolean)** |  |  |  |  |  |
| HF (YES) | 1798 (78.14%) | 1316 (77.59%) | 482 (79.67%) | 0.32 | 0.00 |
| Hypertension (YES) | 1672 (72.66%) | 1239 (73.05%) | 433 (71.57%) | 0.52 | 0.00 |
| AFIB (YES) | **1250 (54.32%)** | **897 (52.89%)** | **353 (58.35%)** | **0.02** | **0.00** |
| T2DM (YES) | 844 (36.68%) | 615 (36.26%) | 229 (37.85%) | 0.52 | 0.00 |
| Renal (YES) | **943 (40.98%)** | **671 (39.56%)** | **272 (44.96%)** | **0.02** | **0.00** |
| Liver (YES) | 88 (3.82%) | 58 (3.42%) | 30 (4.96%) | 0.12 | 0.00 |
| COPD (YES) | 390 (16.95%) | 291 (17.16%) | 99 (16.36%) | 0.7 | 0.00 |
| CAD (YES) | 1366 (59.37%) | 1005 (59.26%) | 361 (59.67%) | 0.9 | 0.00 |
| Stroke (YES) | 227 (9.87%) | 166 (9.79%) | 61 (10.08%) | 0.9 | 0.00 |
| Malignancy (YES) | 28 (1.22%) | 22 (1.30%) | 6 (0.99%) | 0.71 | 0.00 |
| **Vital signs (1st 24 h)** |  |  |  |  |  |
| MAP | 75.00 [65.00, 87.00] | 76.00 [65.00, 87.00] | 75.00 [65.00, 88.00] | 0.8 | 0.04 |
| Temperature | 36.56 [36.27, 36.89] | 36.56 [36.20, 36.89] | 36.56 [36.33, 36.83] | 0.94 | 13.26 |
| Heart rate | 89.00 [77.00, 106.00] | 89.00 [77.75, 106.00] | 88.00 [75.00, 105.00] | 0.31 | 0.00 |
| **Laboratory tests (1st 24 h)** |  |  |  |  |  |
| WBC count | **13.00 [9.30, 18.10]** | **13.30 [9.50, 18.30]** | **12.30 [8.80, 17.20]** | **0.003** | **0.00** |
| Hemoglobin | 10.40 [8.60, 12.20] | 10.40 [8.67, 12.22] | 10.30 [8.50, 12.00] | 0.21 | 0.00 |
| Platelet | 186.00 [128.00, 254.00] | 187.00 [130.00, 253.00] | 184.00 [122.00, 255.00] | 0.6 | 0.00 |
| pH | 7.35 [7.27, 7.41] | 7.35 [7.27, 7.41] | 7.35 [7.27, 7.41] | 0.91 | 7.00 |
| PO2 | 130.00 [78.00, 281.00] | 130.00 [77.00, 281.00] | 131.00 [79.50, 278.50] | 0.87 | 22.95 |
| PCO2 | 40.00 [34.00, 46.00] | 40.00 [34.00, 46.00] | 39.00 [33.00, 46.00] | 0.14 | 25.29 |
| Lactate | 2.40 [1.60, 3.90] | 2.40 [1.60, 3.90] | 2.40 [1.60, 4.10] | 0.65 | 3.61 |
| Creatinine | **1.50 [1.00, 2.40]** | **1.50 [1.00, 2.30]** | **1.70 [1.10, 2.70]** | **<0.001** | **0.00** |
| **Outcomes (boolean)** |  |  |  |  |  |
| 28-day mortality (Death) | **819 (35.59%)** | **504 (29.72%)** | **315 (52.07%)** | **<0.001** | **0.00** |
| ICU mortality (Death) | **618 (26.86%)** | **364 (21.46%)** | **254 (41.98%)** | **<0.001** | **0.00** |
| In-hospital mortality (Death) | **739 (32.12%)** | **453 (26.71%)** | **286 (47.27%)** | **<0.001** | **0.00** |
| **Length of Stay (days)** |  |  |  |  |  |
| ICU LOS | **5.10 [2.92, 9.46]** | **4.85 [2.82, 9.02]** | **6.17 [3.34, 10.52]** | **<0.001** | **0.00** |
| Hospital LOS | 9.93 [5.58, 17.37] | 9.87 [5.90, 17.04] | 10.08 [4.38, 18.06] | 0.21 | 0.00 |
| Values are presented as mean (standard deviation) or median [Q1, Q3] for continuous variables and number (percentage) for categorical variables. Variables in bold have p-value < 0.05. | | | | | |

Supplementary Table 2. Basic demographic characteristics of the eICU cohort

|  | **Overall (N=869)** | **VIS decreasing (N=632)** | **VIS increasing (N=237)** | **p-value** | **Missing data (%)** |
| --- | --- | --- | --- | --- | --- |
| Age | 67.00 [57.00, 76.00] | 67.00 [57.00, 76.00] | 67.00 [58.00, 76.00] | 0.63 | 0.00 |
| Gender (Female) | 321 (36.94%) | 227 (35.92%) | 94 (39.66%) | 0.35 | 0.00 |
| Race |  |  |  |  |  |
| Asian | 8 (0.92%) | 4 (0.63%) | 4 (1.69%) | 0.22 | 0.00 |
| Black | 82 (9.44%) | 54 (8.54%) | 28 (11.81%) |  |  |
| White | 677 (77.91%) | 499 (78.96%) | 178 (75.11%) |  |  |
| Other | 102 (11.74%) | 75 (11.87%) | 27 (11.39%) |  |  |
| Weight | 80.00 [68.50, 99.00] | 80.70 [68.47, 99.70] | 79.40 [69.00, 95.10] | 0.29 | 0.00 |
| SAPS II | 45.00 [34.00, 58.00] | 45.00 [34.00, 58.00] | 46.00 [36.00, 59.00] | 0.24 | 0.00 |
| SOFA score | 10.00 [8.00, 13.00] | 10.00 [8.00, 13.00] | 10.00 [8.00, 13.00] | 0.85 | 0.00 |
| Charlson comorbidity index | 4.00 [2.00, 6.00] | 4.00 [2.00, 6.00] | 4.00 [2.00, 6.00] | 0.18 | 0.00 |
| **Interventions (boolean for 1st 24 h)** |  |  |  |  |  |
| RRT (YES) | 77 (8.86%) | 51 (8.07%) | 26 (10.97%) | 0.23 | 0.00 |
| Mechanical ventilation (YES) | 647 (74.45%) | 461 (72.94%) | 186 (78.48%) | 0.11 | 0.00 |
| Sedative therapy (YES) | 555 (63.87%) | 406 (64.24%) | 149 (62.87%) | 0.77 | 0.00 |
| Albumin (YES) | 89 (10.24%) | 60 (9.49%) | 29 (12.24%) | 0.29 | 0.00 |
| **Comorbidities (boolean)** |  |  |  |  |  |
| HF (YES) | 440 (50.63%) | 307 (48.58%) | 133 (56.12%) | 0.06 | 0.00 |
| Hypertension (YES) | 451 (51.90%) | 316 (50.00%) | 135 (56.96%) | 0.08 | 0.00 |
| AFIB (YES) | 512 (58.92%) | 371 (58.70%) | 141 (59.49%) | 0.89 | 0.00 |
| T2DM (YES) | 321 (36.94%) | 227 (35.92%) | 94 (39.66%) | 0.35 | 0.00 |
| Renal (YES) | **234 (26.93%)** | **153 (24.21%)** | **81 (34.18%)** | **0.004** | **0.00** |
| Liver (YES) | 82 (9.44%) | 64 (10.13%) | 18 (7.59%) | 0.31 | 0.00 |
| COPD (YES) | 164 (18.87%) | 119 (18.83%) | 45 (18.99%) | 1 | 0.00 |
| CAD (YES) | 641 (73.76%) | 462 (73.10%) | 179 (75.53%) | 0.52 | 0.00 |
| Stroke (YES) | 82 (9.44%) | 55 (8.70%) | 27 (11.39%) | 0.28 | 0.00 |
| Malignancy (YES) | 107 (12.31%) | 73 (11.55%) | 34 (14.35%) | 0.32 | 0.00 |
| **Vital signs (1st 24 h)** |  |  |  |  |  |
| MAP | 73.00 [63.00, 86.00] | 73.00 [62.00, 85.00] | 75.00 [64.00, 88.25] | 0.13 | 0.23 |
| Temperature | 36.50 [36.00, 36.90] | 36.50 [35.90, 36.90] | 36.50 [36.15, 36.90] | 0.45 | 0.23 |
| Heart rate | 92.00 [79.00, 111.00] | 92.00 [79.00, 111.00] | 93.00 [79.00, 109.00] | 0.82 | 0.00 |
| **Laboratory tests (1st 24 h)** |  |  |  |  |  |
| WBC count | 13.90 [10.00, 19.20] | 13.60 [10.00, 18.80] | 14.90 [10.10, 20.10] | 0.18 | 1.04 |
| Hemoglobin | 11.20 [9.50, 13.00] | 11.30 [9.60, 13.00] | 11.00 [9.40, 13.10] | 0.77 | 1.04 |
| Platelet | 189.50 [139.00, 246.00] | 191.00 [141.00, 248.00] | 186.00 [134.50, 238.50] | 0.26 | 1.04 |
| pH | **7.32 [7.23, 7.39]** | **7.31 [7.23, 7.38]** | **7.34 [7.24, 7.41]** | **0.04** | **22.67** |
| PO2 | 102.00 [72.00, 174.00] | 101.00 [72.00, 168.00] | 103.00 [72.50, 197.00] | 0.51 | 22.55 |
| PCO2 | 40.05 [33.40, 48.00] | 40.60 [34.00, 48.90] | 39.00 [32.25, 46.85] | 0.09 | 24.28 |
| Lactate | 3.20 [1.70, 6.07] | 3.00 [1.60, 5.90] | 3.70 [1.80, 7.00] | 0.09 | 38.55 |
| Creatinine | **1.60 [1.06, 2.40]** | **1.58 [1.01, 2.30]** | **1.68 [1.15, 2.58]** | **0.03** | **0.23** |
| **Outcomes (boolean)** |  |  |  |  |  |
| ICU mortality (Death) | **228 (26.24%)** | **132 (20.89%)** | **96 (40.51%)** | **<0.001** | **0.00** |
| In-hospital mortality (Death) | **294 (33.83%)** | **178 (28.16%)** | **116 (48.95%)** | **<0.001** | **0.00** |
| **Length of Stay (days)** |  |  |  |  |  |
| ICU LOS | 4.62 [2.69, 8.34] | 4.45 [2.59, 7.90] | 5.02 [2.76, 8.98] | 0.06 | 0.00 |
| Hospital LOS | 7.72 [3.89, 14.42] | 7.77 [4.06, 14.28] | 7.62 [3.23, 15.07] | 0.62 | 0.00 |
| Values are presented as mean (standard deviation) or median [Q1, Q3] for continuous variables and number (percentage) for categorical variables. Variables in bold have p-value < 0.05. | | | | | |

Supplementary Table 3. Unadjusted log-rank test for In-hospital mortality of MIMIC-IV cohort

| Characteristic | HR^1^ | 95% CI^1^ | p-value |
| --- | --- | --- | --- |
| VIS decreasing | 1 | Reference |  |
| VIS increasing | 1.82 | 1.54, 2.14 | <0.001 |
| ^1^HR = Hazard Ratio, CI = Confidence Interval; | | | |

Supplementary Table 4. Unadjusted log-rank test for ICU mortality of MIMIC-IV cohort

| Characteristic | HR^1^ | 95% CI^1^ | p-value |
| --- | --- | --- | --- |
| VIS decreasing | 1 | Reference |  |
| VIS increasing | 1.67 | 1.41, 1.99 | <0.001 |
| ^1^HR = Hazard Ratio, CI = Confidence Interval; | | | |

Supplementary Table 5. Unadjusted log-rank test for 28-day mortality of MIMIC-IV cohort

| Characteristic | HR^1^ | 95% CI^1^ | p-value |
| --- | --- | --- | --- |
| VIS decreasing | 1 | Reference |  |
| VIS increasing | 2.12 | 1.80, 2.50 | <0.001 |
| ^1^HR = Hazard Ratio, CI = Confidence Interval; | | | |

Supplementary Table 6. Unadjusted log-rank test for In-hospital mortality of eICU cohort

| Characteristic | HR^1^ | 95% CI^1^ | p-value |
| --- | --- | --- | --- |
| VIS decreasing | 1 | Reference |  |
| VIS increasing | 1.75 | 1.35, 2.26 | <0.001 |
| ^1^HR = Hazard Ratio, CI = Confidence Interval; | | | |

Supplementary Table 7. Unadjusted log-rank test for ICU mortality of eICU cohort

| Characteristic | HR^1^ | 95% CI^1^ | p-value |
| --- | --- | --- | --- |
| VIS decreasing | 1 | Reference |  |
| VIS increasing | 1.71 | 1.29, 2.28 | <0.001 |
| ^1^HR = Hazard Ratio, CI = Confidence Interval; | | | |

Supplementary Table 8. Multivariate Cox model adjusted with all covariates for In-hospital mortality of MIMIC-IV cohort

| **Characteristic** | **HR** | **95% CI** | **p-value** |
| --- | --- | --- | --- |
| Group |  |  |  |
| VIS decreasing | 1.00 | Reference |  |
| VIS increasing | 1.79 | 1.54, 2.09 | <0.001 |
| Age | 1.02 | 1.01, 1.03 | <0.001 |
| Gender |  |  |  |
| Female | 1.00 | Reference |  |
| Male | 0.94 | 0.80, 1.11 | 0.47 |
| Race |  |  |  |
| Asian | 1.00 | Reference |  |
| Black | 0.91 | 0.52, 1.58 | 0.74 |
| White | 1.37 | 0.84, 2.23 | 0.21 |
| Other | 1.68 | 1.02, 2.75 | 0.04 |
| Weight | 1.00 | 0.99, 1.00 | 0.13 |
| SAPS II | 1.02 | 1.01, 1.03 | <0.001 |
| SOFA score | 1.08 | 1.04, 1.12 | <0.001 |
| Charlson comorbidity index | 1.08 | 1.03, 1.13 | <0.001 |
| RRT |  |  |  |
| YES | 1.00 | Reference |  |
| NO | 0.92 | 0.72, 1.18 | 0.52 |
| Mechanical ventilation |  |  |  |
| YES | 1.00 | Reference |  |
| NO | 0.85 | 0.58, 1.25 | 0.41 |
| Sedative therapy |  |  |  |
| YES | 1.00 | Reference |  |
| NO | 1.87 | 1.27, 2.74 | 0.002 |
| Albumin |  |  |  |
| YES | 1.00 | Reference |  |
| NO | 1.69 | 1.35, 2.12 | <0.001 |
| HF |  |  |  |
| YES | 1.00 | Reference |  |
| NO | 1.53 | 1.27, 1.84 | <0.001 |
| Hypertension |  |  |  |
| YES | 1.00 | Reference |  |
| NO | 1.08 | 0.90, 1.29 | 0.39 |
| AFIB |  |  |  |
| YES | 1.00 | Reference |  |
| NO | 1.27 | 1.09, 1.49 | 0.003 |
| T2DM |  |  |  |
| YES | 1.00 | Reference |  |
| NO | 1.04 | 0.88, 1.23 | 0.63 |
| Renal |  |  |  |
| YES | 1.00 | Reference |  |
| NO | 1.19 | 0.97, 1.47 | 0.1 |
| Liver |  |  |  |
| YES | 1.00 | Reference |  |
| NO | 1.21 | 0.85, 1.73 | 0.28 |
| COPD |  |  |  |
| YES | 1.00 | Reference |  |
| NO | 0.87 | 0.72, 1.04 | 0.13 |
| CAD |  |  |  |
| YES | 1.00 | Reference |  |
| NO | 1.02 | 0.87, 1.20 | 0.81 |
| Stroke |  |  |  |
| YES | 1.00 | Reference |  |
| NO | 1.24 | 0.97, 1.57 | 0.08 |
| Malignancy |  |  |  |
| YES | 1.00 | Reference |  |
| NO | 1.86 | 0.76, 4.57 | 0.17 |
| MAP | 1.00 | 0.99, 1.00 | 0.31 |
| Temperature | 0.95 | 0.89, 1.02 | 0.17 |
| Heart rate | 1.01 | 1.00, 1.01 | <0.001 |
| WBC count | 1.00 | 0.99, 1.01 | 0.9 |
| Hemoglobin | 0.97 | 0.94, 1.00 | 0.09 |
| Platelet | 1.00 | 1.00, 1.00 | 0.28 |
| pH | 0.56 | 0.23, 1.35 | 0.2 |
| PO2 | 1.00 | 1.00, 1.00 | 0.34 |
| PCO2 | 1.00 | 0.99, 1.01 | 0.99 |
| Lactate | 1.05 | 1.02, 1.08 | <0.001 |
| Creatinine | 1.01 | 0.95, 1.07 | 0.74 |
| Abbreviations: CI = Confidence Interval, HR = Hazard Ratio | | | |

Supplementary Table 9. Multivariate Cox model adjusted with covariates selected by uni-variable analyses for In-hospital mortality of MIMIC-IV cohort

| **Characteristic** | **HR** | **95% CI** | **p-value** |
| --- | --- | --- | --- |
| Group |  |  |  |
| VIS decreasing | 1.00 | Reference |  |
| VIS increasing | 1.79 | 1.54, 2.08 | <0.001 |
| Age | 1.01 | 1.01, 1.02 | <0.001 |
| Gender |  |  |  |
| Female | 1.00 | Reference |  |
| Male | 0.94 | 0.80, 1.10 | 0.41 |
| Weight | 1.00 | 0.99, 1.00 | 0.13 |
| SAPS II | 1.02 | 1.01, 1.03 | <0.001 |
| SOFA score | 1.04 | 1.01, 1.08 | 0.007 |
| Charlson comorbidity index | 1.07 | 1.03, 1.12 | <0.001 |
| RRT |  |  |  |
| YES | 1.00 | Reference |  |
| NO | 0.89 | 0.70, 1.13 | 0.33 |
| Albumin |  |  |  |
| YES | 1.00 | Reference |  |
| NO | 1.80 | 1.44, 2.25 | <0.001 |
| HF |  |  |  |
| YES | 1.00 | Reference |  |
| NO | 1.51 | 1.26, 1.80 | <0.001 |
| T2DM |  |  |  |
| YES | 1.00 | Reference |  |
| NO | 1.03 | 0.88, 1.21 | 0.73 |
| Renal |  |  |  |
| YES | 1.00 | Reference |  |
| NO | 1.15 | 0.95, 1.41 | 0.16 |
| COPD |  |  |  |
| YES | 1.00 | Reference |  |
| NO | 0.84 | 0.70, 1.01 | 0.07 |
| Temperature | 0.94 | 0.88, 1.01 | 0.08 |
| Heart rate | 1.01 | 1.00, 1.01 | <0.001 |
| Hemoglobin | 0.97 | 0.94, 1.00 | 0.1 |
| pH | 0.63 | 0.30, 1.36 | 0.24 |
| PO2 | 1.00 | 1.00, 1.00 | 0.12 |
| Lactate | 1.04 | 1.02, 1.07 | 0.001 |
| Creatinine | 1.02 | 0.97, 1.08 | 0.46 |
| Abbreviations: CI = Confidence Interval, HR = Hazard Ratio | | | |

Supplementary Table 10. Multivariate Cox model adjusted with covariates selected by Boruta algorithm for In-hospital mortality of MIMIC-IV cohort

| **Characteristic** | **HR** | **95% CI** | **p-value** |
| --- | --- | --- | --- |
| Group |  |  |  |
| VIS decreasing | 1.00 | Reference |  |
| VIS increasing | 1.79 | 1.54, 2.08 | <0.001 |
| Creatinine | 0.98 | 0.93, 1.03 | 0.45 |
| Lactate | 1.04 | 1.01, 1.07 | 0.006 |
| PCO2 | 1.00 | 0.99, 1.01 | 0.74 |
| PO2 | 1.00 | 1.00, 1.00 | 0.35 |
| pH | 0.44 | 0.19, 1.06 | 0.07 |
| Platelet | 1.00 | 1.00, 1.00 | 0.28 |
| Hemoglobin | 0.97 | 0.94, 1.00 | 0.07 |
| WBC count | 1.00 | 0.99, 1.01 | 0.98 |
| Heart rate | 1.01 | 1.00, 1.01 | 0.002 |
| Temperature | 0.96 | 0.89, 1.02 | 0.18 |
| Stroke |  |  |  |
| YES | 1.00 | Reference |  |
| NO | 1.18 | 0.94, 1.49 | 0.16 |
| HF |  |  |  |
| YES | 1.00 | Reference |  |
| NO | 1.62 | 1.35, 1.94 | <0.001 |
| Albumin |  |  |  |
| YES | 1.00 | Reference |  |
| NO | 1.69 | 1.36, 2.12 | <0.001 |
| Sedative therapy |  |  |  |
| YES | 1.00 | Reference |  |
| NO | 1.87 | 1.28, 2.75 | 0.001 |
| Mechanical ventilation |  |  |  |
| YES | 1.00 | Reference |  |
| NO | 0.85 | 0.58, 1.24 | 0.39 |
| RRT |  |  |  |
| YES | 1.00 | Reference |  |
| NO | 0.87 | 0.69, 1.11 | 0.27 |
| Charlson comorbidity index | 1.06 | 1.03, 1.10 | <0.001 |
| SOFA score | 1.07 | 1.04, 1.11 | <0.001 |
| SAPS II | 1.02 | 1.02, 1.03 | <0.001 |
| Weight | 1.00 | 0.99, 1.00 | 0.06 |
| Age | 1.01 | 1.01, 1.02 | <0.001 |
| Abbreviations: CI = Confidence Interval, HR = Hazard Ratio | | | |

Supplementary Table 11. Multivariate Cox model adjusted with all covariates for ICU mortality of MIMIC-IV cohort

| **Characteristic** | **HR** | **95% CI** | **p-value** |
| --- | --- | --- | --- |
| Group |  |  |  |
| VIS decreasing | 1.00 | Reference |  |
| VIS increasing | 1.69 | 1.43, 2.00 | <0.001 |
| Age | 1.01 | 1.01, 1.02 | 0.001 |
| Gender |  |  |  |
| Female | 1.00 | Reference |  |
| Male | 1.01 | 0.85, 1.21 | 0.9 |
| Race |  |  |  |
| Asian | 1.00 | Reference |  |
| Black | 1.01 | 0.57, 1.81 | 0.97 |
| White | 1.22 | 0.72, 2.05 | 0.47 |
| Other | 1.50 | 0.88, 2.55 | 0.14 |
| Weight | 1.00 | 0.99, 1.00 | 0.09 |
| SAPS II | 1.02 | 1.01, 1.03 | <0.001 |
| SOFA score | 1.06 | 1.02, 1.10 | 0.006 |
| Charlson comorbidity index | 1.08 | 1.03, 1.14 | 0.002 |
| RRT |  |  |  |
| YES | 1.00 | Reference |  |
| NO | 0.88 | 0.68, 1.14 | 0.33 |
| Mechanical ventilation |  |  |  |
| YES | 1.00 | Reference |  |
| NO | 0.83 | 0.54, 1.28 | 0.4 |
| Sedative therapy |  |  |  |
| YES | 1.00 | Reference |  |
| NO | 2.06 | 1.33, 3.21 | 0.001 |
| Albumin |  |  |  |
| YES | 1.00 | Reference |  |
| NO | 1.71 | 1.35, 2.17 | <0.001 |
| HF |  |  |  |
| YES | 1.00 | Reference |  |
| NO | 1.40 | 1.14, 1.71 | 0.001 |
| Hypertension |  |  |  |
| YES | 1.00 | Reference |  |
| NO | 1.03 | 0.85, 1.26 | 0.74 |
| AFIB |  |  |  |
| YES | 1.00 | Reference |  |
| NO | 1.35 | 1.13, 1.60 | <0.001 |
| T2DM |  |  |  |
| YES | 1.00 | Reference |  |
| NO | 1.06 | 0.88, 1.27 | 0.54 |
| Renal |  |  |  |
| YES | 1.00 | Reference |  |
| NO | 1.28 | 1.02, 1.61 | 0.03 |
| Liver |  |  |  |
| YES | 1.00 | Reference |  |
| NO | 1.26 | 0.84, 1.89 | 0.26 |
| COPD |  |  |  |
| YES | 1.00 | Reference |  |
| NO | 0.91 | 0.74, 1.13 | 0.4 |
| CAD |  |  |  |
| YES | 1.00 | Reference |  |
| NO | 0.98 | 0.82, 1.17 | 0.84 |
| Stroke |  |  |  |
| YES | 1.00 | Reference |  |
| NO | 1.44 | 1.10, 1.87 | 0.008 |
| Malignancy |  |  |  |
| YES | 1.00 | Reference |  |
| NO | 1.97 | 0.72, 5.37 | 0.18 |
| MAP | 1.00 | 0.99, 1.00 | 0.6 |
| Temperature | 0.98 | 0.91, 1.05 | 0.55 |
| Heart rate | 1.00 | 1.00, 1.01 | 0.01 |
| WBC count | 1.00 | 0.99, 1.01 | 0.69 |
| Hemoglobin | 0.98 | 0.94, 1.01 | 0.24 |
| Platelet | 1.00 | 1.00, 1.00 | 0.84 |
| pH | 0.27 | 0.10, 0.72 | 0.008 |
| PO2 | 1.00 | 1.00, 1.00 | 0.48 |
| PCO2 | 1.00 | 0.99, 1.00 | 0.42 |
| Lactate | 1.05 | 1.02, 1.08 | 0.002 |
| Creatinine | 1.01 | 0.95, 1.08 | 0.73 |
| Abbreviations: CI = Confidence Interval, HR = Hazard Ratio | | | |

Supplementary Table 12. Multivariate Cox model adjusted with covariates selected by uni-variable analyses for ICU mortality of MIMIC-IV cohort

| **Characteristic** | **HR** | **95% CI** | **p-value** |
| --- | --- | --- | --- |
| Group |  |  |  |
| VIS decreasing | 1.00 | Reference |  |
| VIS increasing | 1.68 | 1.42, 1.98 | <0.001 |
| Age | 1.01 | 1.00, 1.02 | 0.002 |
| Gender |  |  |  |
| Female | 1.00 | Reference |  |
| Male | 0.99 | 0.83, 1.18 | 0.92 |
| Weight | 1.00 | 0.99, 1.00 | 0.08 |
| SAPS II | 1.02 | 1.01, 1.03 | <0.001 |
| SOFA score | 1.02 | 0.99, 1.06 | 0.21 |
| Charlson comorbidity index | 1.07 | 1.02, 1.12 | 0.006 |
| RRT |  |  |  |
| YES | 1.00 | Reference |  |
| NO | 0.83 | 0.64, 1.07 | 0.15 |
| Albumin |  |  |  |
| YES | 1.00 | Reference |  |
| NO | 1.82 | 1.44, 2.30 | <0.001 |
| HF |  |  |  |
| YES | 1.00 | Reference |  |
| NO | 1.36 | 1.12, 1.65 | 0.002 |
| T2DM |  |  |  |
| YES | 1.00 | Reference |  |
| NO | 1.02 | 0.85, 1.22 | 0.82 |
| Renal |  |  |  |
| YES | 1.00 | Reference |  |
| NO | 1.22 | 0.98, 1.52 | 0.08 |
| COPD |  |  |  |
| YES | 1.00 | Reference |  |
| NO | 0.90 | 0.73, 1.11 | 0.32 |
| Temperature | 0.96 | 0.90, 1.03 | 0.28 |
| Heart rate | 1.00 | 1.00, 1.01 | 0.01 |
| Hemoglobin | 0.98 | 0.95, 1.01 | 0.25 |
| pH | 0.37 | 0.16, 0.85 | 0.02 |
| PO2 | 1.00 | 1.00, 1.00 | 0.25 |
| Lactate | 1.05 | 1.02, 1.08 | 0.001 |
| Creatinine | 1.03 | 0.97, 1.09 | 0.38 |
| Abbreviations: CI = Confidence Interval, HR = Hazard Ratio | | | |

Supplementary Table 13. Multivariate Cox model adjusted with covariates selected by Boruta algorithm for ICU mortality of MIMIC-IV cohort

| **Characteristic** | **HR** | **95% CI** | **p-value** |
| --- | --- | --- | --- |
| Group |  |  |  |
| VIS decreasing | 1.00 | Reference |  |
| VIS increasing | 1.68 | 1.42, 1.98 | <0.001 |
| Creatinine | 0.98 | 0.92, 1.04 | 0.51 |
| Lactate | 1.04 | 1.01, 1.07 | 0.009 |
| PCO2 | 1.00 | 0.99, 1.00 | 0.22 |
| PO2 | 1.00 | 1.00, 1.00 | 0.45 |
| pH | 0.21 | 0.08, 0.55 | 0.001 |
| Platelet | 1.00 | 1.00, 1.00 | 0.78 |
| Hemoglobin | 0.98 | 0.95, 1.01 | 0.24 |
| WBC count | 1.00 | 0.99, 1.01 | 0.75 |
| Heart rate | 1.00 | 1.00, 1.01 | 0.02 |
| Temperature | 0.98 | 0.91, 1.05 | 0.54 |
| Stroke |  |  |  |
| YES | 1.00 | Reference |  |
| NO | 1.35 | 1.04, 1.75 | 0.02 |
| HF |  |  |  |
| YES | 1.00 | Reference |  |
| NO | 1.46 | 1.20, 1.78 | <0.001 |
| Albumin |  |  |  |
| YES | 1.00 | Reference |  |
| NO | 1.72 | 1.36, 2.18 | <0.001 |
| Sedative therapy |  |  |  |
| YES | 1.00 | Reference |  |
| NO | 2.04 | 1.32, 3.17 | 0.001 |
| Mechanical ventilation |  |  |  |
| YES | 1.00 | Reference |  |
| NO | 0.81 | 0.52, 1.24 | 0.33 |
| RRT |  |  |  |
| YES | 1.00 | Reference |  |
| NO | 0.84 | 0.65, 1.08 | 0.16 |
| Charlson comorbidity index | 1.05 | 1.01, 1.09 | 0.01 |
| SOFA score | 1.05 | 1.01, 1.09 | 0.02 |
| SAPS II | 1.02 | 1.01, 1.03 | <0.001 |
| Weight | 1.00 | 0.99, 1.00 | 0.06 |
| Age | 1.01 | 1.00, 1.02 | 0.004 |
| Abbreviations: CI = Confidence Interval, HR = Hazard Ratio | | | |

Supplementary Table 14. Multivariate Cox model adjusted with all covariates for 28-day mortality of MIMIC-IV cohort

| **Characteristic** | **HR** | **95% CI** | **p-value** |
| --- | --- | --- | --- |
| Group |  |  |  |
| VIS decreasing | 1.00 | Reference |  |
| VIS increasing | 2.00 | 1.73, 2.32 | <0.001 |
| Age | 1.02 | 1.01, 1.03 | <0.001 |
| Gender |  |  |  |
| Female | 1.00 | Reference |  |
| Male | 0.95 | 0.81, 1.10 | 0.49 |
| Race |  |  |  |
| Asian | 1.00 | Reference |  |
| Black | 1.29 | 0.76, 2.17 | 0.35 |
| White | 1.66 | 1.03, 2.67 | 0.04 |
| Other | 2.02 | 1.25, 3.27 | 0.004 |
| Weight | 1.00 | 0.99, 1.00 | 0.09 |
| SAPS II | 1.02 | 1.01, 1.03 | <0.001 |
| SOFA score | 1.10 | 1.06, 1.14 | <0.001 |
| Charlson comorbidity index | 1.09 | 1.04, 1.14 | <0.001 |
| RRT |  |  |  |
| YES | 1.00 | Reference |  |
| NO | 0.86 | 0.68, 1.09 | 0.21 |
| Mechanical ventilation |  |  |  |
| YES | 1.00 | Reference |  |
| NO | 0.94 | 0.66, 1.34 | 0.73 |
| Sedative therapy |  |  |  |
| YES | 1.00 | Reference |  |
| NO | 1.73 | 1.21, 2.48 | 0.003 |
| Albumin |  |  |  |
| YES | 1.00 | Reference |  |
| NO | 1.91 | 1.52, 2.41 | <0.001 |
| HF |  |  |  |
| YES | 1.00 | Reference |  |
| NO | 1.41 | 1.18, 1.68 | <0.001 |
| Hypertension |  |  |  |
| YES | 1.00 | Reference |  |
| NO | 1.11 | 0.94, 1.32 | 0.21 |
| AFIB |  |  |  |
| YES | 1.00 | Reference |  |
| NO | 1.12 | 0.96, 1.30 | 0.15 |
| T2DM |  |  |  |
| YES | 1.00 | Reference |  |
| NO | 1.06 | 0.90, 1.24 | 0.48 |
| Renal |  |  |  |
| YES | 1.00 | Reference |  |
| NO | 1.14 | 0.93, 1.39 | 0.2 |
| Liver |  |  |  |
| YES | 1.00 | Reference |  |
| NO | 1.17 | 0.83, 1.65 | 0.37 |
| COPD |  |  |  |
| YES | 1.00 | Reference |  |
| NO | 0.88 | 0.74, 1.05 | 0.17 |
| CAD |  |  |  |
| YES | 1.00 | Reference |  |
| NO | 1.08 | 0.92, 1.26 | 0.33 |
| Stroke |  |  |  |
| YES | 1.00 | Reference |  |
| NO | 1.14 | 0.90, 1.44 | 0.26 |
| Malignancy |  |  |  |
| YES | 1.00 | Reference |  |
| NO | 1.23 | 0.61, 2.49 | 0.57 |
| MAP | 1.00 | 1.00, 1.00 | 0.79 |
| Temperature | 0.94 | 0.88, 1.00 | 0.05 |
| Heart rate | 1.01 | 1.00, 1.01 | <0.001 |
| WBC count | 1.00 | 0.99, 1.01 | 0.98 |
| Hemoglobin | 0.95 | 0.92, 0.98 | 0.004 |
| Platelet | 1.00 | 1.00, 1.00 | 0.04 |
| pH | 0.72 | 0.31, 1.68 | 0.45 |
| PO2 | 1.00 | 1.00, 1.00 | 0.19 |
| PCO2 | 1.00 | 1.00, 1.01 | 0.7 |
| Lactate | 1.05 | 1.02, 1.08 | <0.001 |
| Creatinine | 1.00 | 0.95, 1.06 | 0.92 |
| Abbreviations: CI = Confidence Interval, HR = Hazard Ratio | | | |

Supplementary Table 15. Multivariate Cox model adjusted with covariates selected by uni-variable analyses for 28-day mortality of MIMIC-IV cohort

| **Characteristic** | **HR** | **95% CI** | **p-value** |
| --- | --- | --- | --- |
| Group |  |  |  |
| VIS decreasing | 1.00 | Reference |  |
| VIS increasing | 2.02 | 1.75, 2.34 | <0.001 |
| Age | 1.02 | 1.01, 1.02 | <0.001 |
| Gender |  |  |  |
| Female | 1.00 | Reference |  |
| Male | 0.93 | 0.80, 1.08 | 0.33 |
| Weight | 1.00 | 0.99, 1.00 | 0.12 |
| SAPS II | 1.02 | 1.01, 1.03 | <0.001 |
| SOFA score | 1.05 | 1.02, 1.09 | <0.001 |
| Charlson comorbidity index | 1.08 | 1.04, 1.12 | <0.001 |
| RRT |  |  |  |
| YES | 1.00 | Reference |  |
| NO | 0.84 | 0.66, 1.06 | 0.13 |
| Albumin |  |  |  |
| YES | 1.00 | Reference |  |
| NO | 2.07 | 1.65, 2.60 | <0.001 |
| HF |  |  |  |
| YES | 1.00 | Reference |  |
| NO | 1.40 | 1.18, 1.66 | <0.001 |
| T2DM |  |  |  |
| YES | 1.00 | Reference |  |
| NO | 1.06 | 0.91, 1.24 | 0.46 |
| Renal |  |  |  |
| YES | 1.00 | Reference |  |
| NO | 1.11 | 0.92, 1.34 | 0.27 |
| COPD |  |  |  |
| YES | 1.00 | Reference |  |
| NO | 0.86 | 0.72, 1.03 | 0.1 |
| Temperature | 0.93 | 0.87, 0.99 | 0.03 |
| Heart rate | 1.01 | 1.00, 1.01 | <0.001 |
| Hemoglobin | 0.96 | 0.93, 0.99 | 0.01 |
| pH | 0.81 | 0.39, 1.68 | 0.57 |
| PO2 | 1.00 | 1.00, 1.00 | 0.03 |
| Lactate | 1.05 | 1.02, 1.07 | <0.001 |
| Creatinine | 1.02 | 0.97, 1.07 | 0.52 |
| Abbreviations: CI = Confidence Interval, HR = Hazard Ratio | | | |

Supplementary Table 16. Multivariate Cox model adjusted with covariates selected by Boruta algorithm for 28-day mortality of MIMIC-IV cohort

| **Characteristic** | **HR** | **95% CI** | **p-value** |
| --- | --- | --- | --- |
| Group |  |  |  |
| VIS decreasing | 1.00 | Reference |  |
| VIS increasing | 2.03 | 1.76, 2.34 | <0.001 |
| Creatinine | 0.98 | 0.93, 1.03 | 0.39 |
| Lactate | 1.04 | 1.02, 1.07 | 0.001 |
| PCO2 | 1.00 | 1.0, 1.01 | 0.8 |
| PO2 | 1.00 | 1.00, 1.00 | 0.15 |
| pH | 0.66 | 0.28, 1.52 | 0.33 |
| Platelet | 1.00 | 1.00, 1.00 | 0.05 |
| Hemoglobin | 0.96 | 0.93, 0.99 | 0.009 |
| WBC count | 1.00 | 0.99, 1.01 | 0.94 |
| Heart rate | 1.01 | 1.00, 1.01 | <0.001 |
| Temperature | 0.93 | 0.87, 1.00 | 0.04 |
| Stroke |  |  |  |
| YES | 1.00 | Reference |  |
| NO | 1.10 | 0.88, 1.39 | 0.4 |
| HF |  |  |  |
| YES | 1.00 | Reference |  |
| NO | 1.49 | 1.25, 1.77 | <0.001 |
| Albumin |  |  |  |
| YES | 1.00 | Reference |  |
| NO | 1.92 | 1.52, 2.41 | <0.001 |
| Sedative therapy |  |  |  |
| YES | 1.00 | Reference |  |
| NO | 1.79 | 1.25, 2.55 | 0.001 |
| Mechanical ventilation |  |  |  |
| YES | 1.00 | Reference |  |
| NO | 0.91 | 0.64, 1.29 | 0.59 |
| RRT |  |  |  |
| YES | 1.00 | Reference |  |
| NO | 0.84 | 0.66, 1.05 | 0.13 |
| Charlson comorbidity index | 1.07 | 1.03, 1.11 | <0.001 |
| SOFA score | 1.09 | 1.06, 1.13 | <0.001 |
| SAPS II | 1.02 | 1.01, 1.03 | <0.001 |
| Weight | 1.00 | 0.99, 1.00 | 0.04 |
| Age | 1.02 | 1.01, 1.02 | <0.001 |
| Abbreviations: CI = Confidence Interval, HR = Hazard Ratio | | | |

Supplementary Table 17. Multivariate Cox model adjusted with all covariates for In-hospital mortality of eICU cohort

| **Characteristic** | **HR** | **95% CI** | **p-value** |
| --- | --- | --- | --- |
| Group |  |  |  |
| VIS decreasing | 1.00 | Reference |  |
| VIS increasing | 1.79 | 1.39, 2.30 | <0.001 |
| Age | 1.01 | 0.99, 1.02 | 0.37 |
| Gender |  |  |  |
| Female | 1.00 | Reference |  |
| Male | 1.06 | 0.81, 1.38 | 0.67 |
| Race |  |  |  |
| Asian | 1.00 | Reference |  |
| Black | 0.33 | 0.13, 0.87 | 0.02 |
| White | 0.32 | 0.13, 0.76 | 0.01 |
| Other | 0.35 | 0.14, 0.88 | 0.03 |
| Weight | 1.00 | 1.00, 1.01 | 0.6 |
| SAPS II | 1.03 | 1.02, 1.04 | <0.001 |
| SOFA score | 1.05 | 0.99, 1.12 | 0.08 |
| Charlson comorbidity index | 1.11 | 1.00, 1.22 | 0.05 |
| RRT |  |  |  |
| YES | 1.00 | Reference |  |
| NO | 0.72 | 0.48, 1.07 | 0.11 |
| Mechanical ventilation |  |  |  |
| YES | 1.00 | Reference |  |
| NO | 0.41 | 0.26, 0.67 | <0.001 |
| Sedative therapy |  |  |  |
| YES | 1.00 | Reference |  |
| NO | 2.34 | 1.67, 3.28 | <0.001 |
| Albumin |  |  |  |
| YES | 1.00 | Reference |  |
| NO | 1.00 | 0.69, 1.44 | 0.99 |
| HF |  |  |  |
| YES | 1.00 | Reference |  |
| NO | 1.01 | 0.77, 1.33 | 0.93 |
| Hypertension |  |  |  |
| YES | 1.00 | Reference |  |
| NO | 1.17 | 0.90, 1.52 | 0.23 |
| AFIB |  |  |  |
| YES | 1.00 | Reference |  |
| NO | 0.90 | 0.70, 1.17 | 0.43 |
| T2DM |  |  |  |
| YES | 1.00 | Reference |  |
| NO | 0.99 | 0.75, 1.31 | 0.95 |
| Renal |  |  |  |
| YES | 1.00 | Reference |  |
| NO | 1.72 | 1.19, 2.50 | 0.004 |
| Liver |  |  |  |
| YES | 1.00 | Reference |  |
| NO | 0.77 | 0.53, 1.12 | 0.17 |
| COPD |  |  |  |
| YES | 1.00 | Reference |  |
| NO | 0.79 | 0.58, 1.08 | 0.14 |
| CAD |  |  |  |
| YES | 1.00 | Reference |  |
| NO | 1.10 | 0.82, 1.48 | 0.53 |
| Stroke |  |  |  |
| YES | 1.00 | Reference |  |
| NO | 0.99 | 0.67, 1.47 | 0.96 |
| Malignancy |  |  |  |
| YES | 1.00 | Reference |  |
| NO | 1.13 | 0.73, 1.73 | 0.59 |
| MAP | 0.99 | 0.99, 1.00 | 0.03 |
| Temperature | 0.89 | 0.84, 0.96 | 0.001 |
| Heart rate | 1.00 | 1.00, 1.01 | 0.08 |
| WBC count | 1.01 | 1.00, 1.02 | 0.26 |
| Hemoglobin | 1.04 | 0.99, 1.10 | 0.13 |
| Platelet | 1.00 | 1.00, 1.00 | 0.76 |
| pH | 0.20 | 0.06, 0.68 | 0.01 |
| PO2 | 1.00 | 1.00, 1.00 | 0.29 |
| PCO2 | 0.98 | 0.97, 1.0 | 0.004 |
| Lactate | 1.03 | 1.00, 1.07 | 0.09 |
| Creatinine | 1.03 | 0.94, 1.14 | 0.5 |
| Abbreviations: CI = Confidence Interval, HR = Hazard Ratio | | | |

Supplementary Table 18. Multivariate Cox model adjusted with covariates selected by uni-variable analyses for In-hospital mortality of eICU cohort

| **Characteristic** | **HR** | **95% CI** | **p-value** |
| --- | --- | --- | --- |
| Group |  |  |  |
| VIS decreasing | 1.00 | Reference |  |
| VIS increasing | 1.87 | 1.46, 2.39 | <0.001 |
| Age | 1.01 | 1.00, 1.02 | 0.007 |
| Race |  |  |  |
| Asian | 1.00 | Reference |  |
| Black | 0.25 | 0.10, 0.62 | 0.003 |
| White | 0.24 | 0.10, 0.55 | <0.001 |
| Other | 0.28 | 0.11, 0.67 | 0.005 |
| SAPS II | 1.03 | 1.01, 1.04 | <0.001 |
| SOFA score | 1.02 | 0.97, 1.08 | 0.35 |
| RRT |  |  |  |
| YES | 1.00 | Reference |  |
| NO | 0.76 | 0.52, 1.11 | 0.16 |
| Mechanical ventilation |  |  |  |
| YES | 1.00 | Reference |  |
| NO | 0.65 | 0.41, 1.02 | 0.06 |
| Hypertension |  |  |  |
| YES | 1.00 | Reference |  |
| NO | 1.21 | 0.95, 1.54 | 0.13 |
| Liver |  |  |  |
| YES | 1.00 | Reference |  |
| NO | 0.78 | 0.55, 1.12 | 0.18 |
| CAD |  |  |  |
| YES | 1.00 | Reference |  |
| NO | 1.08 | 0.83, 1.41 | 0.57 |
| MAP | 0.99 | 0.99, 1.00 | 0.04 |
| Temperature | 0.90 | 0.85, 0.96 | 0.001 |
| WBC count | 1.01 | 1.00, 1.01 | 0.31 |
| pH | 0.23 | 0.07, 0.76 | 0.02 |
| PCO2 | 0.98 | 0.97, 1.0 | 0.003 |
| Lactate | 1.03 | 0.99, 1.06 | 0.13 |
| Creatinine | 1.00 | 0.92, 1.09 | 0.92 |
| Abbreviations: CI = Confidence Interval, HR = Hazard Ratio | | | |

Supplementary Table 19. Multivariate Cox model adjusted with covariates selected by Boruta algorithm for In-hospital mortality of eICU cohort

| **Characteristic** | **HR** | **95% CI** | **p-value** |
| --- | --- | --- | --- |
| Group |  |  |  |
| VIS decreasing | 1.00 | Reference |  |
| VIS increasing | 1.75 | 1.37, 2.24 | <0.001 |
| Creatinine | 0.96 | 0.88, 1.05 | 0.37 |
| Lactate | 1.03 | 1.00, 1.06 | 0.07 |
| PCO2 | 0.98 | 0.97, 1.0 | 0.004 |
| pH | 0.21 | 0.06, 0.68 | 0.009 |
| Temperature | 0.91 | 0.86, 0.97 | 0.006 |
| MAP | 0.99 | 0.99, 1.00 | 0.04 |
| Sedative therapy |  |  |  |
| YES | 1.00 | Reference |  |
| NO | 2.19 | 1.59, 3.00 | <0.001 |
| Mechanical ventilation |  |  |  |
| YES | 1.00 | Reference |  |
| NO | 0.41 | 0.26, 0.66 | <0.001 |
| RRT |  |  |  |
| YES | 1.00 | Reference |  |
| NO | 0.79 | 0.54, 1.16 | 0.24 |
| Charlson comorbidity index | 1.05 | 1.00, 1.10 | 0.04 |
| SOFA score | 1.04 | 0.99, 1.09 | 0.13 |
| SAPS II | 1.03 | 1.02, 1.04 | <0.001 |
| Abbreviations: CI = Confidence Interval, HR = Hazard Ratio | | | |

Supplementary Table 20. Multivariate Cox model adjusted with all covariates for ICU mortality of eICU cohort

| **Characteristic** | **HR** | **95% CI** | **p-value** |
| --- | --- | --- | --- |
| Group |  |  |  |
| VIS decreasing | 1.00 | Reference |  |
| VIS increasing | 1.92 | 1.44, 2.55 | <0.001 |
| Age | 1.00 | 0.99, 1.02 | 0.79 |
| Gender |  |  |  |
| Female | 1.00 | Reference |  |
| Male | 1.04 | 0.77, 1.41 | 0.79 |
| Race |  |  |  |
| Asian | 1.00 | Reference |  |
| Black | 0.31 | 0.11, 0.92 | 0.03 |
| White | 0.35 | 0.13, 0.92 | 0.03 |
| Other | 0.35 | 0.12, 0.97 | 0.04 |
| Weight | 1.00 | 1.00, 1.01 | 0.52 |
| SAPS II | 1.03 | 1.02, 1.04 | <0.001 |
| SOFA score | 1.04 | 0.98, 1.11 | 0.22 |
| Charlson comorbidity index | 1.14 | 1.01, 1.27 | 0.03 |
| RRT |  |  |  |
| YES | 1.00 | Reference |  |
| NO | 0.83 | 0.52, 1.31 | 0.42 |
| Mechanical ventilation |  |  |  |
| YES | 1.00 | Reference |  |
| NO | 0.41 | 0.22, 0.77 | 0.005 |
| Sedative therapy |  |  |  |
| YES | 1.00 | Reference |  |
| NO | 2.26 | 1.54, 3.32 | <0.001 |
| Albumin |  |  |  |
| YES | 1.00 | Reference |  |
| NO | 0.92 | 0.62, 1.37 | 0.68 |
| HF |  |  |  |
| YES | 1.00 | Reference |  |
| NO | 1.04 | 0.77, 1.42 | 0.78 |
| Hypertension |  |  |  |
| YES | 1.00 | Reference |  |
| NO | 1.42 | 1.05, 1.91 | 0.02 |
| AFIB |  |  |  |
| YES | 1.00 | Reference |  |
| NO | 0.93 | 0.69, 1.25 | 0.62 |
| T2DM |  |  |  |
| YES | 1.00 | Reference |  |
| NO | 1.08 | 0.78, 1.50 | 0.64 |
| Renal |  |  |  |
| YES | 1.00 | Reference |  |
| NO | 1.80 | 1.16, 2.79 | 0.009 |
| Liver |  |  |  |
| YES | 1.00 | Reference |  |
| NO | 0.67 | 0.45, 0.99 | 0.05 |
| COPD |  |  |  |
| YES | 1.00 | Reference |  |
| NO | 0.95 | 0.66, 1.38 | 0.79 |
| CAD |  |  |  |
| YES | 1.00 | Reference |  |
| NO | 1.07 | 0.76, 1.50 | 0.71 |
| Stroke |  |  |  |
| YES | 1.00 | Reference |  |
| NO | 0.99 | 0.62, 1.57 | 0.96 |
| Malignancy |  |  |  |
| YES | 1.00 | Reference |  |
| NO | 1.31 | 0.80, 2.15 | 0.29 |
| MAP | 0.99 | 0.99, 1.00 | 0.09 |
| Temperature | 0.93 | 0.86, 1.01 | 0.08 |
| Heart rate | 1.00 | 1.00, 1.01 | 0.2 |
| WBC count | 1.00 | 0.99, 1.02 | 0.5 |
| Hemoglobin | 1.04 | 0.98, 1.10 | 0.21 |
| Platelet | 1.00 | 1.00, 1.00 | 0.65 |
| pH | 0.11 | 0.03, 0.42 | 0.001 |
| PO2 | 1.00 | 1.00, 1.00 | 0.12 |
| PCO2 | 0.98 | 0.97, 1.00 | 0.01 |
| Lactate | 1.03 | 1.0, 1.07 | 0.09 |
| Creatinine | 1.08 | 0.97, 1.21 | 0.17 |
| Abbreviations: CI = Confidence Interval, HR = Hazard Ratio | | | |

Supplementary Table 21. Multivariate Cox model adjusted with covariates selected by uni-variable analyses for ICU mortality of eICU cohort

| **Characteristic** | **HR** | **95% CI** | **p-value** |
| --- | --- | --- | --- |
| Group |  |  |  |
| VIS decreasing | 1.00 | Reference |  |
| VIS increasing | 1.95 | 1.47, 2.57 | <0.001 |
| Age | 1.01 | 1.00, 1.02 | 0.05 |
| Race |  |  |  |
| Asian | 1.00 | Reference |  |
| Black | 0.23 | 0.08, 0.65 | 0.005 |
| White | 0.27 | 0.11, 0.68 | 0.005 |
| Other | 0.29 | 0.11, 0.77 | 0.01 |
| SAPS II | 1.03 | 1.02, 1.04 | <0.001 |
| SOFA score | 1.02 | 0.96, 1.08 | 0.61 |
| RRT |  |  |  |
| YES | 1.00 | Reference |  |
| NO | 0.85 | 0.55, 1.31 | 0.46 |
| Mechanical ventilation |  |  |  |
| YES | 1.00 | Reference |  |
| NO | 0.60 | 0.33, 1.10 | 0.1 |
| Hypertension |  |  |  |
| YES | 1.00 | Reference |  |
| NO | 1.45 | 1.10, 1.91 | 0.009 |
| Liver |  |  |  |
| YES | 1.00 | Reference |  |
| NO | 0.68 | 0.47, 0.99 | 0.04 |
| CAD |  |  |  |
| YES | 1.00 | Reference |  |
| NO | 1.05 | 0.77, 1.43 | 0.74 |
| MAP | 0.99 | 0.99, 1.00 | 0.06 |
| Temperature | 0.93 | 0.86, 1.00 | 0.05 |
| WBC count | 1.00 | 0.99, 1.02 | 0.46 |
| pH | 0.11 | 0.03, 0.42 | 0.001 |
| PCO2 | 0.98 | 0.97, 0.99 | 0.003 |
| Lactate | 1.03 | 0.99, 1.07 | 0.13 |
| Creatinine | 1.04 | 0.94, 1.14 | 0.44 |
| Abbreviations: CI = Confidence Interval, HR = Hazard Ratio | | | |

Supplementary Table 22. Multivariate Cox model adjusted with covariates selected by Boruta algorithm for ICU mortality of eICU cohort

| **Characteristic** | **HR** | **95% CI** | **p-value** |
| --- | --- | --- | --- |
| Group |  |  |  |
| VIS decreasing | 1.00 | Reference |  |
| VIS increasing | 1.82 | 1.38, 2.41 | <0.001 |
| Creatinine | 0.99 | 0.90, 1.09 | 0.87 |
| Lactate | 1.03 | 0.99, 1.06 | 0.12 |
| PCO2 | 0.98 | 0.97, 0.99 | 0.004 |
| pH | 0.11 | 0.03, 0.40 | <0.001 |
| Temperature | 0.95 | 0.88, 1.03 | 0.22 |
| MAP | 0.99 | 0.99, 1.00 | 0.06 |
| Sedative therapy |  |  |  |
| YES | 1.00 | Reference |  |
| NO | 2.12 | 1.48, 3.04 | <0.001 |
| Mechanical ventilation |  |  |  |
| YES | 1.00 | Reference |  |
| NO | 0.40 | 0.22, 0.74 | 0.004 |
| RRT |  |  |  |
| YES | 1.00 | Reference |  |
| NO | 0.89 | 0.58, 1.39 | 0.62 |
| Charlson comorbidity index | 1.04 | 0.98, 1.10 | 0.2 |
| SOFA score | 1.03 | 0.97, 1.09 | 0.3 |
| SAPS II | 1.04 | 1.02, 1.05 | <0.001 |
| Abbreviations: CI = Confidence Interval, HR = Hazard Ratio | | | |

Supplementary Table 23. Machine learning model performance in the MIMIC-IV cohort

| **Model** | **AUC** | **Accuracy** | **Recall** | **TNR** | **PPV** | **F-beta score** | **NPV** | **FNR** | **FPR** | **BACC** | **CE** | **DOR** | **MCC** |
| --- | --- | --- | --- | --- | --- | --- | --- | --- | --- | --- | --- | --- | --- |
| AdaBoost | 0.73 | 0.69 | 0.43 | 0.84 | 0.61 | 0.50 | 0.71 | 0.57 | 0.16 | 0.63 | 0.63 | 3.85 | 0.29 |
| CatBoost | 0.73 | 0.69 | 0.40 | 0.85 | 0.61 | 0.48 | 0.71 | 0.60 | 0.15 | 0.63 | 0.58 | 3.88 | 0.29 |
| LightGBM | 0.72 | 0.70 | 0.39 | 0.88 | 0.65 | 0.49 | 0.71 | 0.61 | 0.12 | 0.63 | 0.59 | 4.58 | 0.31 |
| XGBoost | 0.72 | 0.69 | 0.45 | 0.83 | 0.61 | 0.52 | 0.72 | 0.55 | 0.17 | 0.64 | 0.58 | 4.16 | 0.31 |
| Random Forest | 0.72 | 0.69 | 0.39 | 0.86 | 0.62 | 0.48 | 0.71 | 0.61 | 0.14 | 0.63 | 0.58 | 3.89 | 0.29 |
| Extra Trees | 0.71 | 0.68 | 0.37 | 0.86 | 0.62 | 0.47 | 0.70 | 0.63 | 0.14 | 0.62 | 0.59 | 3.82 | 0.28 |
| SVM | 0.71 | 0.66 | 0.30 | 0.86 | 0.56 | 0.39 | 0.68 | 0.70 | 0.14 | 0.58 | 0.64 | 2.70 | 0.20 |
| SGD Classifier | 0.71 | 0.69 | 0.48 | 0.82 | 0.61 | 0.53 | 0.73 | 0.52 | 0.18 | 0.65 | 0.99 | 4.15 | 0.32 |
| Gradient Boosting | 0.71 | 0.68 | 0.39 | 0.85 | 0.59 | 0.47 | 0.70 | 0.61 | 0.15 | 0.62 | 0.60 | 3.47 | 0.26 |
| Neural Network | 0.70 | 0.68 | 0.45 | 0.82 | 0.59 | 0.51 | 0.72 | 0.55 | 0.18 | 0.64 | 0.65 | 3.74 | 0.29 |
| Logistic Regression | 0.70 | 0.67 | 0.34 | 0.86 | 0.59 | 0.43 | 0.69 | 0.66 | 0.14 | 0.60 | 0.63 | 3.23 | 0.24 |
| Naive Bayes | 0.67 | 0.65 | 0.35 | 0.82 | 0.53 | 0.42 | 0.68 | 0.65 | 0.18 | 0.58 | 1.29 | 2.43 | 0.19 |

Supplementary Table 24. Machine learning model performance in the eICU cohort

| **Model** | **AUC** | **Accuracy** | **Recall** | **TNR** | **PPV** | **F-beta score** | **NPV** | **FNR** | **FPR** | **BACC** | **CE** | **DOR** | **MCC** |
| --- | --- | --- | --- | --- | --- | --- | --- | --- | --- | --- | --- | --- | --- |
| LightGBM | 0.69 | 0.65 | 0.43 | 0.80 | 0.60 | 0.50 | 0.66 | 0.57 | 0.20 | 0.62 | 0.62 | 3.01 | 0.25 |
| Gradient Boosting | 0.69 | 0.65 | 0.47 | 0.77 | 0.60 | 0.53 | 0.67 | 0.53 | 0.23 | 0.62 | 0.62 | 3.05 | 0.26 |
| CatBoost | 0.68 | 0.65 | 0.45 | 0.79 | 0.60 | 0.52 | 0.67 | 0.55 | 0.21 | 0.62 | 0.63 | 3.06 | 0.26 |
| Random Forest | 0.68 | 0.64 | 0.43 | 0.79 | 0.59 | 0.50 | 0.66 | 0.57 | 0.21 | 0.61 | 0.62 | 2.81 | 0.23 |
| XGBoost | 0.68 | 0.63 | 0.49 | 0.72 | 0.56 | 0.52 | 0.67 | 0.51 | 0.28 | 0.61 | 0.63 | 2.52 | 0.22 |
| AdaBoost | 0.68 | 0.64 | 0.48 | 0.75 | 0.58 | 0.52 | 0.67 | 0.52 | 0.25 | 0.61 | 0.65 | 2.76 | 0.24 |
| Neural Network | 0.66 | 0.66 | 0.56 | 0.73 | 0.60 | 0.58 | 0.70 | 0.44 | 0.27 | 0.64 | 0.79 | 3.44 | 0.29 |
| SVM | 0.65 | 0.64 | 0.45 | 0.77 | 0.58 | 0.51 | 0.66 | 0.55 | 0.23 | 0.61 | 0.79 | 2.75 | 0.23 |
| Logistic Regression | 0.64 | 0.64 | 0.45 | 0.77 | 0.58 | 0.50 | 0.66 | 0.55 | 0.23 | 0.61 | 0.76 | 2.69 | 0.23 |
| Extra Trees | 0.64 | 0.61 | 0.41 | 0.76 | 0.55 | 0.47 | 0.64 | 0.59 | 0.24 | 0.58 | 0.66 | 2.17 | 0.18 |
| SGD Classifier | 0.64 | 0.61 | 0.52 | 0.68 | 0.54 | 0.53 | 0.67 | 0.48 | 0.32 | 0.60 | 1.26 | 2.29 | 0.20 |
| Naive Bayes | 0.59 | 0.61 | 0.41 | 0.75 | 0.54 | 0.47 | 0.64 | 0.59 | 0.25 | 0.58 | 3.39 | 2.09 | 0.17 |
