# Supplementary figures and images for: Vasoactive–Inotropic Score Reduction Rate Is Highly Associated With Prognosis for Critically Ill Patients With Cardiogenic Shock: Insights From the Real‐World Dynamic Data
Source: Cardiovasc Ther. 2026 Mar 6;2026:8989505. doi: 10.1155/cdr/8989505 (PMC12966616; doi:10.1155/cdr/8989505)

**A**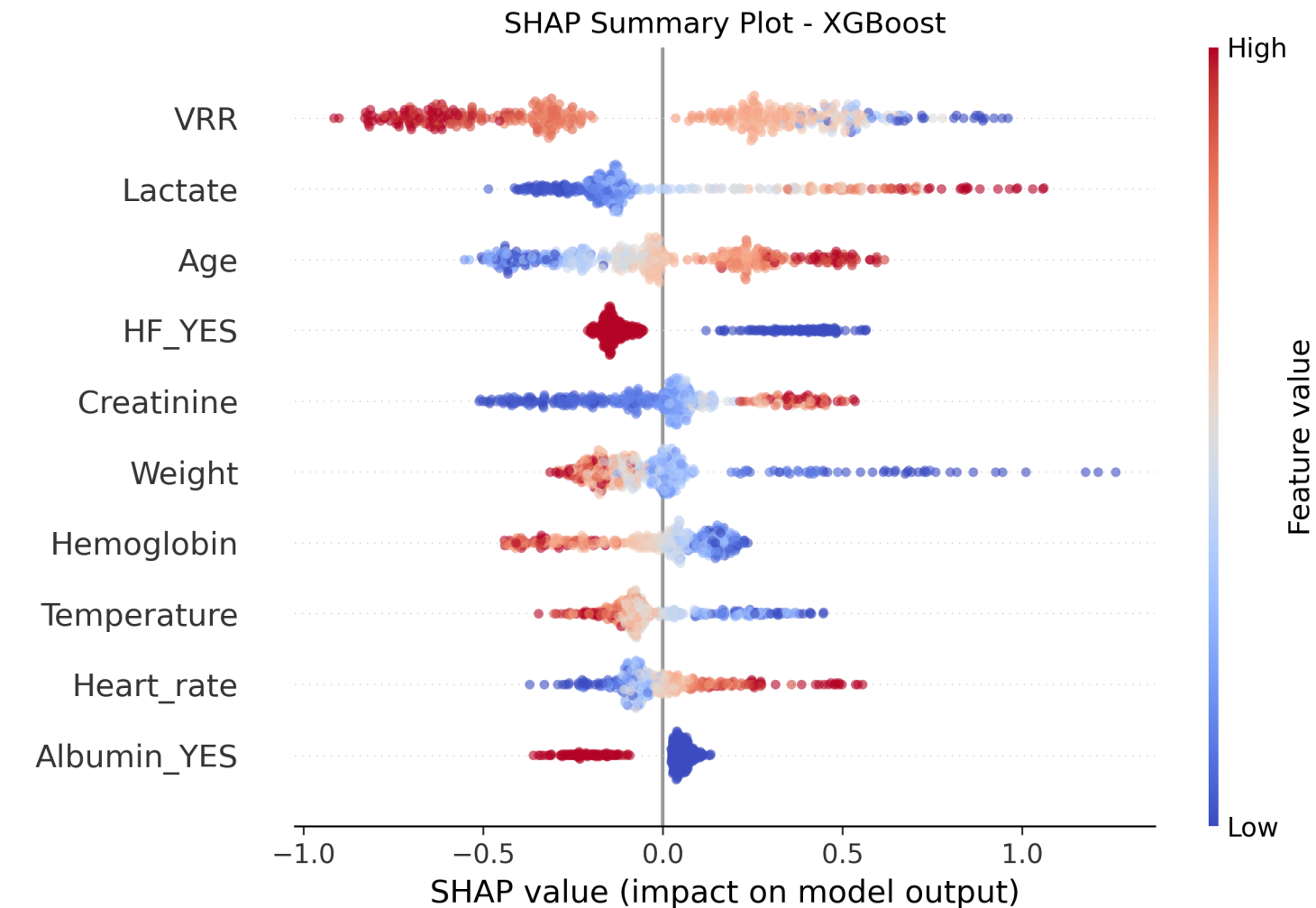**B**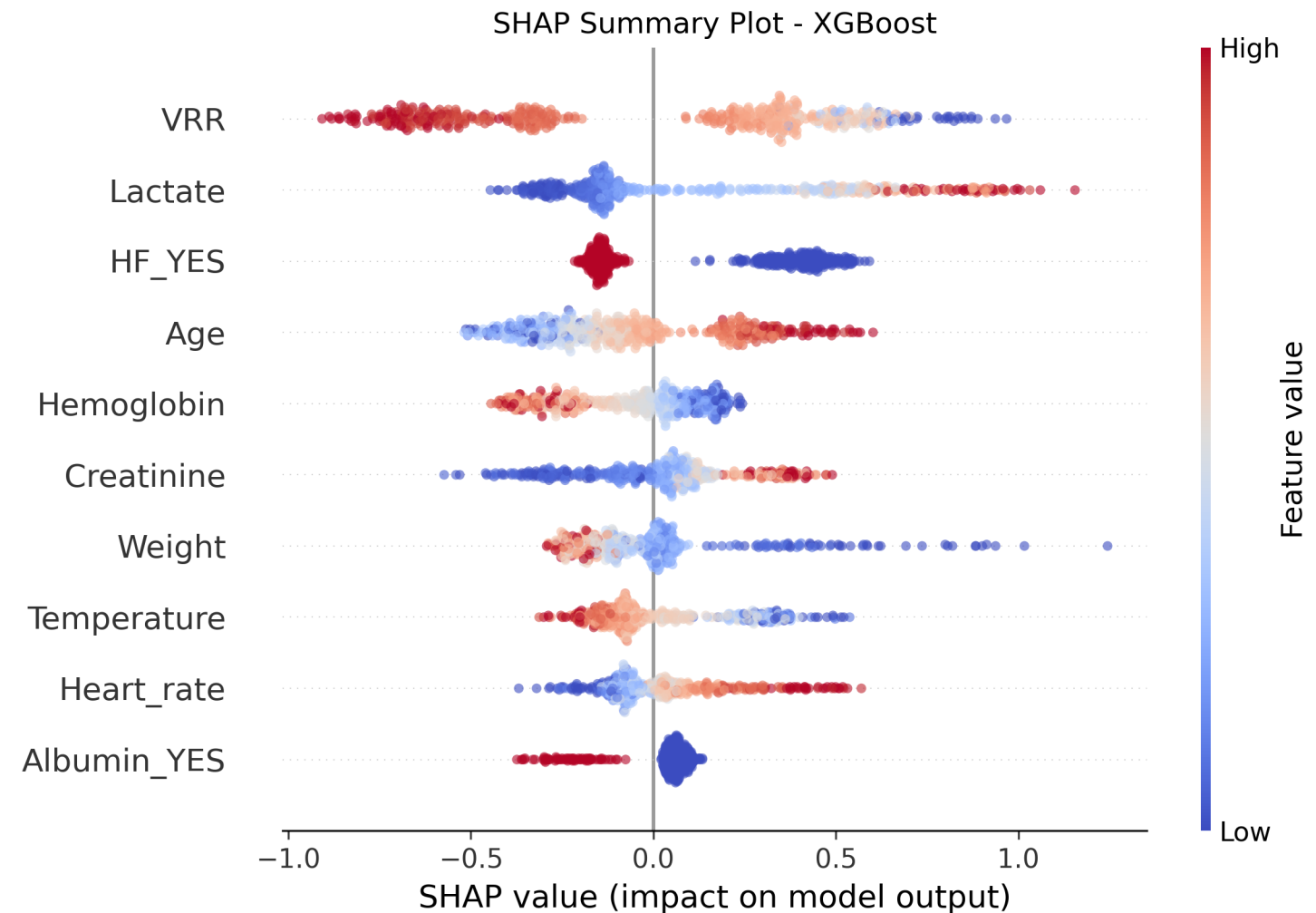

Supplement: Supplementary file 1 — Supporting Information Additional supporting information can be found online in the Supporting Information section. Figure S1 Unadjusted Kaplan–Meier curve for secondary outcome. (A) Kaplan–Meier curve of ICU mortality for the MIMIC‐IV cohort. (B) Kaplan–Meier curve of ICU mortality for the eICU cohort. Figure S2: Unadjusted Kaplan–Meier curve for 28‐day outcome. Figure S3: Feature selection results using the Boruta algorithm. (A) Relative importance of candidate predictors for in‐hospital mortality for the MIMIC‐IV cohort. (B) Relative importance of candidate predictors for in‐hospital mortality for the eICU cohort. Figure S4: Forest plot of subgroup analysis. (A) Forest plot of subgroup analysis of ICU mortality for the MIMIC‐IV cohort. (B) Forest plot of subgroup analysis of ICU mortality for the eICU cohort. Figure S5: Forest plot of subgroup analysis of 28‐day mortality for the MIMIC‐IV cohort. Figure S6: SHAP (SHapley Additive exPlanations) summary plots showing the contribution of the top features to the LightGBM model predictions. (A) SHAP summary plots of the LightGBM model for the MIMIC‐IV cohort. (B) SHAP summary plots of the LightGBM model for the eICU cohort. Figure S7: SHAP (SHapley Additive exPlanations) summary plots showing the contribution of the top features to the XGBoost model predictions. (A) SHAP summary plots of the XGBoost model for the MIMIC‐IV cohort. (B) SHAP summary plots of the XGBoost model for the eICU cohort. Table S1: Basic demographic characteristics of the MIMIC‐IV cohort. Table S2: Basic demographic characteristics of the eICU cohort. Table S3: Unadjusted log‐rank test for in‐hospital mortality of the MIMIC‐IV cohort. Table S4: Unadjusted log‐rank test for ICU mortality of the MIMIC‐IV cohort. Table S5: Unadjusted log‐rank test for 28‐day mortality of the MIMIC‐IV cohort. Table S6: Unadjusted log‐rank test for in‐hospital mortality of the eICU cohort. Table S7: Unadjusted log‐rank test for ICU mortality of the eICU cohort. Table [file CDR-2026-8989505-s001.zip › Sypplementary Figure 7.pdf]

**A**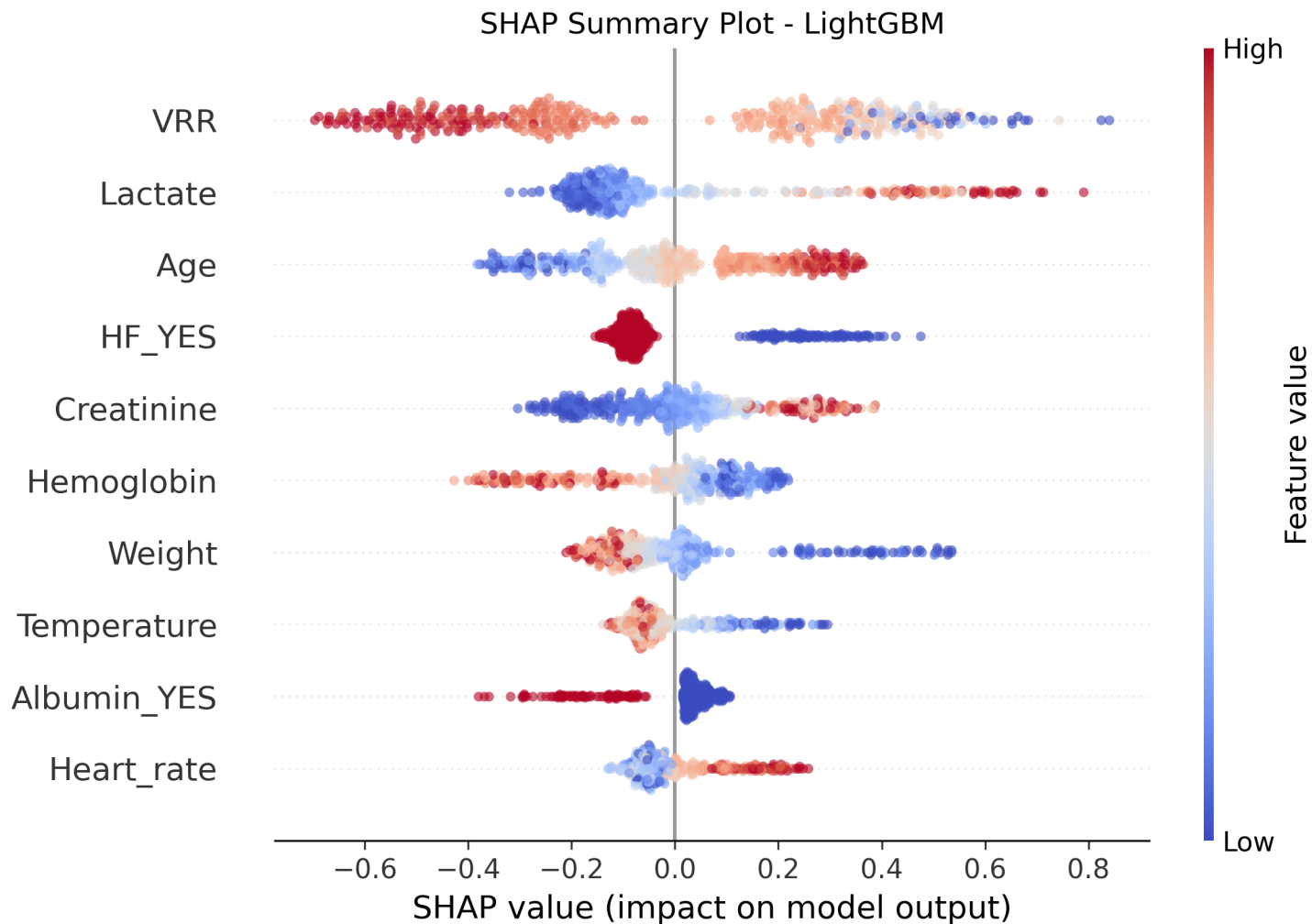**B**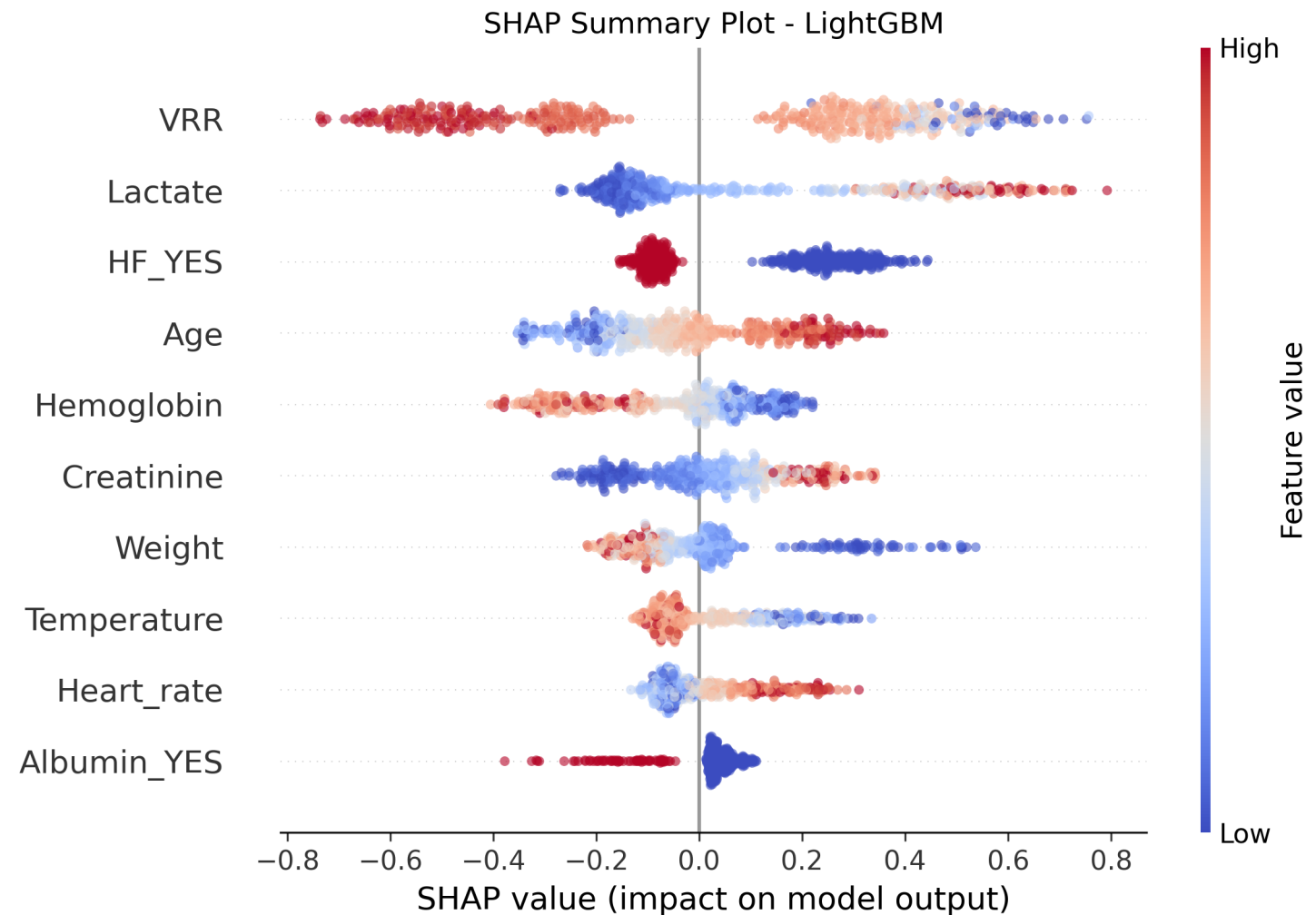

Supplement: Supplementary file 1 — Supporting Information Additional supporting information can be found online in the Supporting Information section. Figure S1 Unadjusted Kaplan–Meier curve for secondary outcome. (A) Kaplan–Meier curve of ICU mortality for the MIMIC‐IV cohort. (B) Kaplan–Meier curve of ICU mortality for the eICU cohort. Figure S2: Unadjusted Kaplan–Meier curve for 28‐day outcome. Figure S3: Feature selection results using the Boruta algorithm. (A) Relative importance of candidate predictors for in‐hospital mortality for the MIMIC‐IV cohort. (B) Relative importance of candidate predictors for in‐hospital mortality for the eICU cohort. Figure S4: Forest plot of subgroup analysis. (A) Forest plot of subgroup analysis of ICU mortality for the MIMIC‐IV cohort. (B) Forest plot of subgroup analysis of ICU mortality for the eICU cohort. Figure S5: Forest plot of subgroup analysis of 28‐day mortality for the MIMIC‐IV cohort. Figure S6: SHAP (SHapley Additive exPlanations) summary plots showing the contribution of the top features to the LightGBM model predictions. (A) SHAP summary plots of the LightGBM model for the MIMIC‐IV cohort. (B) SHAP summary plots of the LightGBM model for the eICU cohort. Figure S7: SHAP (SHapley Additive exPlanations) summary plots showing the contribution of the top features to the XGBoost model predictions. (A) SHAP summary plots of the XGBoost model for the MIMIC‐IV cohort. (B) SHAP summary plots of the XGBoost model for the eICU cohort. Table S1: Basic demographic characteristics of the MIMIC‐IV cohort. Table S2: Basic demographic characteristics of the eICU cohort. Table S3: Unadjusted log‐rank test for in‐hospital mortality of the MIMIC‐IV cohort. Table S4: Unadjusted log‐rank test for ICU mortality of the MIMIC‐IV cohort. Table S5: Unadjusted log‐rank test for 28‐day mortality of the MIMIC‐IV cohort. Table S6: Unadjusted log‐rank test for in‐hospital mortality of the eICU cohort. Table S7: Unadjusted log‐rank test for ICU mortality of the eICU cohort. Table [file CDR-2026-8989505-s001.zip › Sypplementary Figure 6.pdf]

A

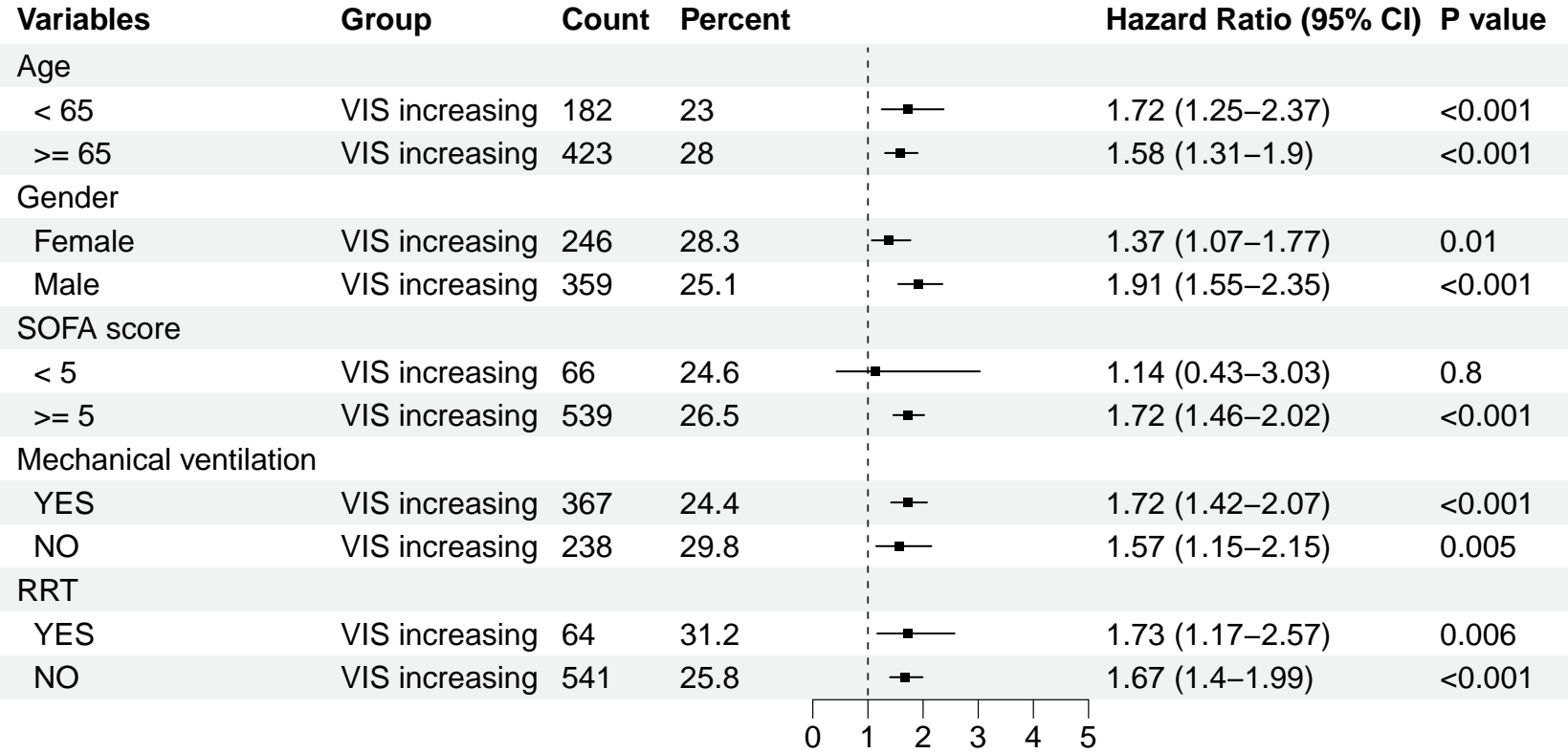

B

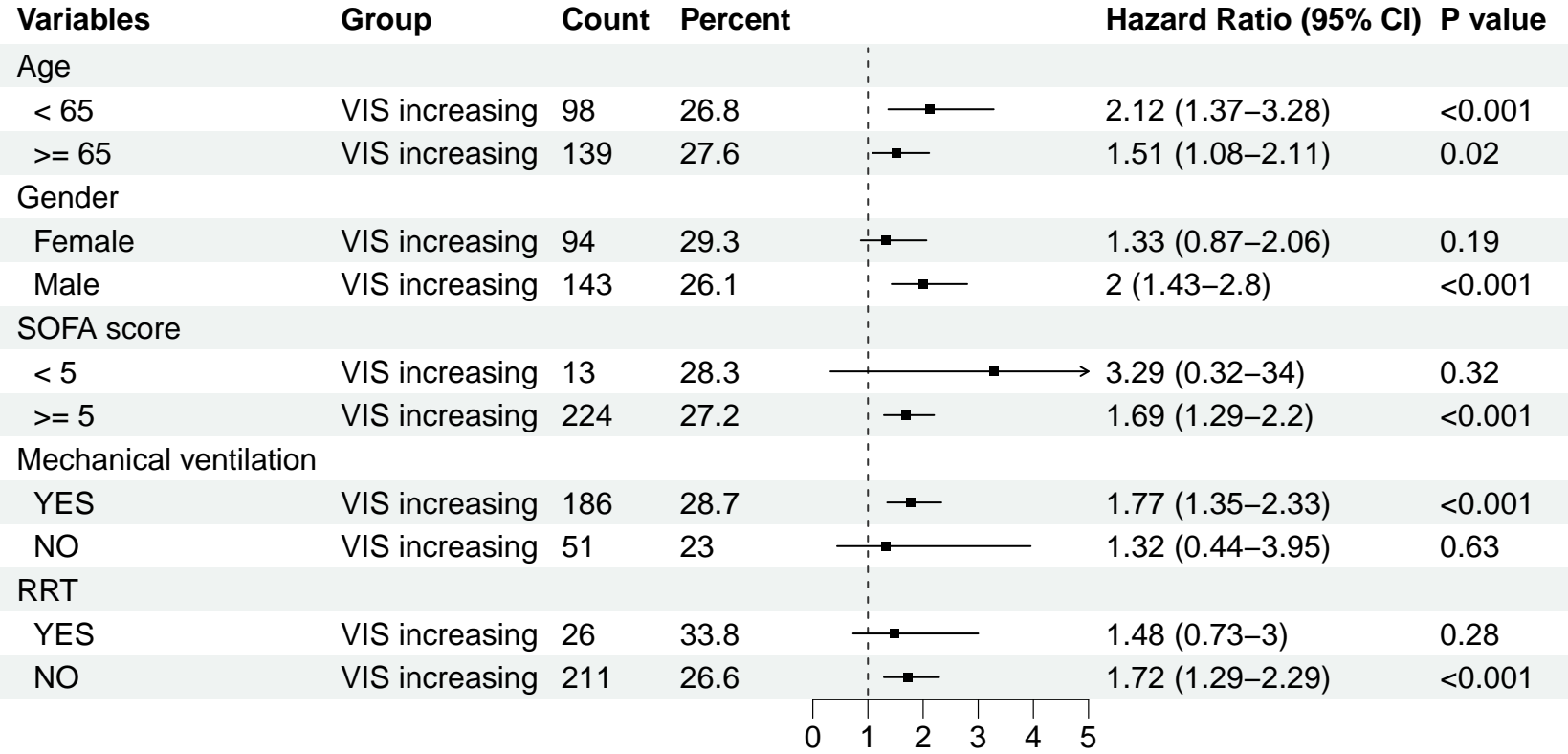

Supplement: Supplementary file 1 — Supporting Information Additional supporting information can be found online in the Supporting Information section. Figure S1 Unadjusted Kaplan–Meier curve for secondary outcome. (A) Kaplan–Meier curve of ICU mortality for the MIMIC‐IV cohort. (B) Kaplan–Meier curve of ICU mortality for the eICU cohort. Figure S2: Unadjusted Kaplan–Meier curve for 28‐day outcome. Figure S3: Feature selection results using the Boruta algorithm. (A) Relative importance of candidate predictors for in‐hospital mortality for the MIMIC‐IV cohort. (B) Relative importance of candidate predictors for in‐hospital mortality for the eICU cohort. Figure S4: Forest plot of subgroup analysis. (A) Forest plot of subgroup analysis of ICU mortality for the MIMIC‐IV cohort. (B) Forest plot of subgroup analysis of ICU mortality for the eICU cohort. Figure S5: Forest plot of subgroup analysis of 28‐day mortality for the MIMIC‐IV cohort. Figure S6: SHAP (SHapley Additive exPlanations) summary plots showing the contribution of the top features to the LightGBM model predictions. (A) SHAP summary plots of the LightGBM model for the MIMIC‐IV cohort. (B) SHAP summary plots of the LightGBM model for the eICU cohort. Figure S7: SHAP (SHapley Additive exPlanations) summary plots showing the contribution of the top features to the XGBoost model predictions. (A) SHAP summary plots of the XGBoost model for the MIMIC‐IV cohort. (B) SHAP summary plots of the XGBoost model for the eICU cohort. Table S1: Basic demographic characteristics of the MIMIC‐IV cohort. Table S2: Basic demographic characteristics of the eICU cohort. Table S3: Unadjusted log‐rank test for in‐hospital mortality of the MIMIC‐IV cohort. Table S4: Unadjusted log‐rank test for ICU mortality of the MIMIC‐IV cohort. Table S5: Unadjusted log‐rank test for 28‐day mortality of the MIMIC‐IV cohort. Table S6: Unadjusted log‐rank test for in‐hospital mortality of the eICU cohort. Table S7: Unadjusted log‐rank test for ICU mortality of the eICU cohort. Table [file CDR-2026-8989505-s001.zip › Sypplementary Figure 4.pdf]

**A**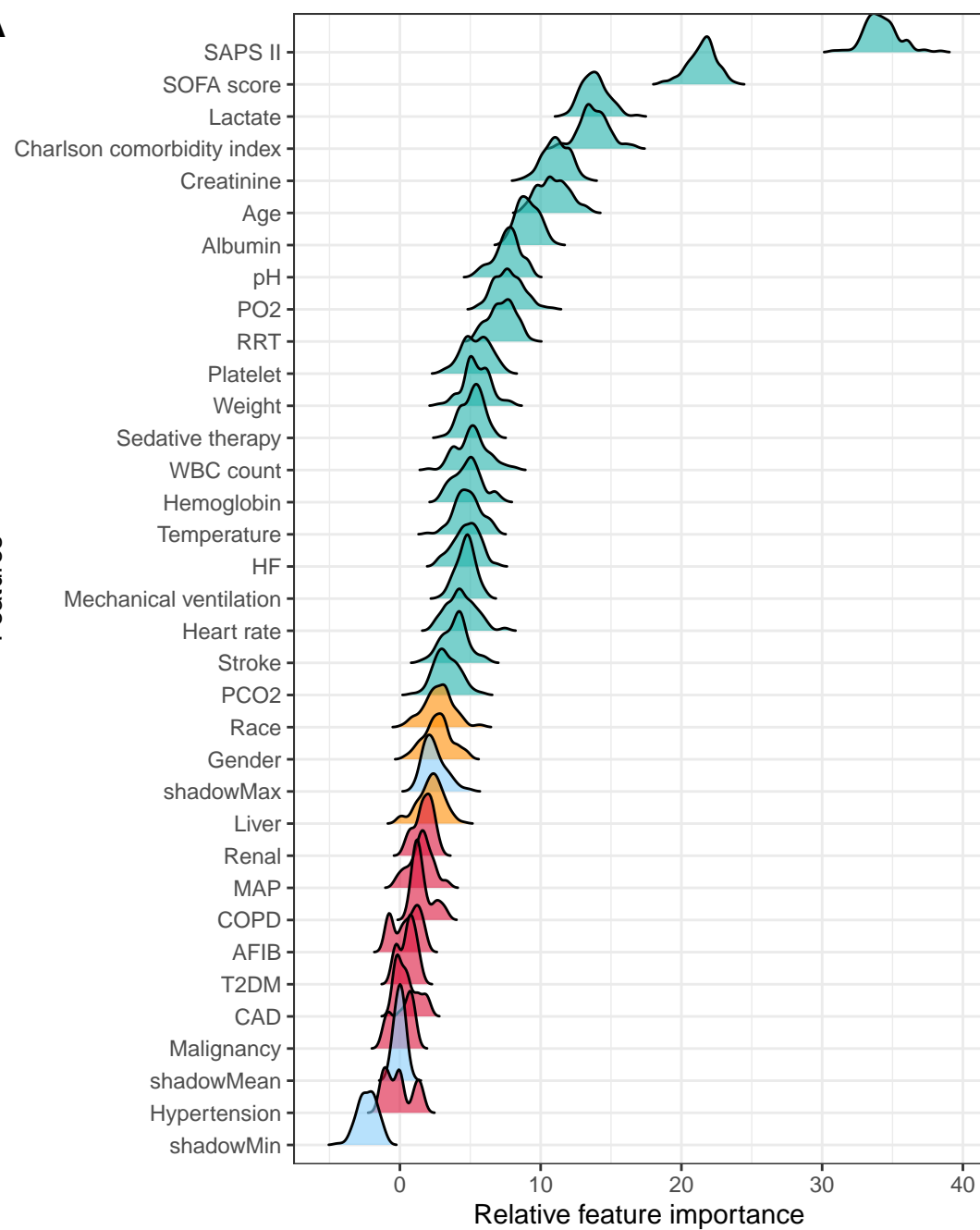**B**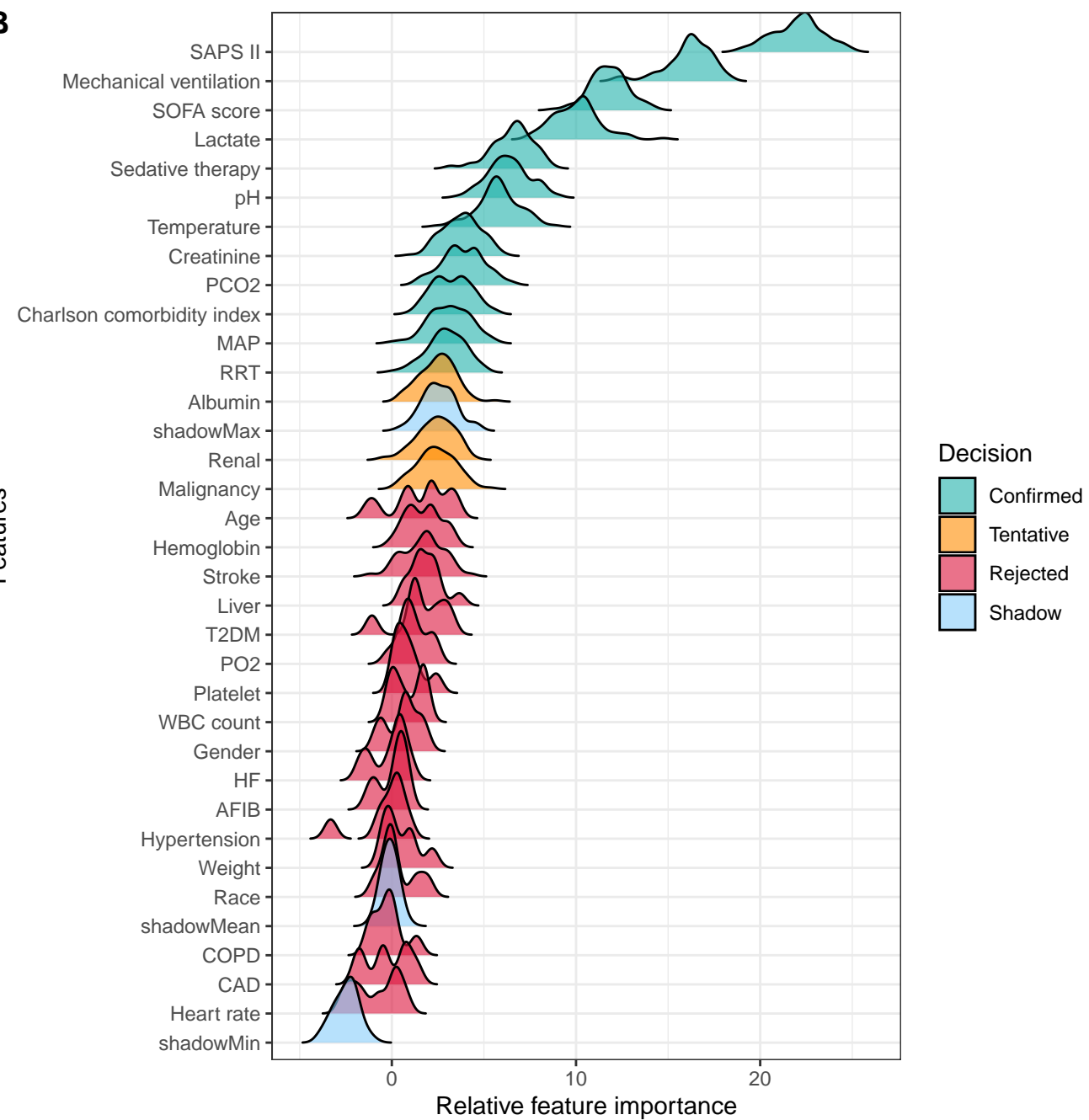

Supplement: Supplementary file 1 — Supporting Information Additional supporting information can be found online in the Supporting Information section. Figure S1 Unadjusted Kaplan–Meier curve for secondary outcome. (A) Kaplan–Meier curve of ICU mortality for the MIMIC‐IV cohort. (B) Kaplan–Meier curve of ICU mortality for the eICU cohort. Figure S2: Unadjusted Kaplan–Meier curve for 28‐day outcome. Figure S3: Feature selection results using the Boruta algorithm. (A) Relative importance of candidate predictors for in‐hospital mortality for the MIMIC‐IV cohort. (B) Relative importance of candidate predictors for in‐hospital mortality for the eICU cohort. Figure S4: Forest plot of subgroup analysis. (A) Forest plot of subgroup analysis of ICU mortality for the MIMIC‐IV cohort. (B) Forest plot of subgroup analysis of ICU mortality for the eICU cohort. Figure S5: Forest plot of subgroup analysis of 28‐day mortality for the MIMIC‐IV cohort. Figure S6: SHAP (SHapley Additive exPlanations) summary plots showing the contribution of the top features to the LightGBM model predictions. (A) SHAP summary plots of the LightGBM model for the MIMIC‐IV cohort. (B) SHAP summary plots of the LightGBM model for the eICU cohort. Figure S7: SHAP (SHapley Additive exPlanations) summary plots showing the contribution of the top features to the XGBoost model predictions. (A) SHAP summary plots of the XGBoost model for the MIMIC‐IV cohort. (B) SHAP summary plots of the XGBoost model for the eICU cohort. Table S1: Basic demographic characteristics of the MIMIC‐IV cohort. Table S2: Basic demographic characteristics of the eICU cohort. Table S3: Unadjusted log‐rank test for in‐hospital mortality of the MIMIC‐IV cohort. Table S4: Unadjusted log‐rank test for ICU mortality of the MIMIC‐IV cohort. Table S5: Unadjusted log‐rank test for 28‐day mortality of the MIMIC‐IV cohort. Table S6: Unadjusted log‐rank test for in‐hospital mortality of the eICU cohort. Table S7: Unadjusted log‐rank test for ICU mortality of the eICU cohort. Table [file CDR-2026-8989505-s001.zip › Sypplementary Figure 3.pdf]

Group    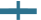 VIS decreasing    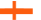 VIS increasing

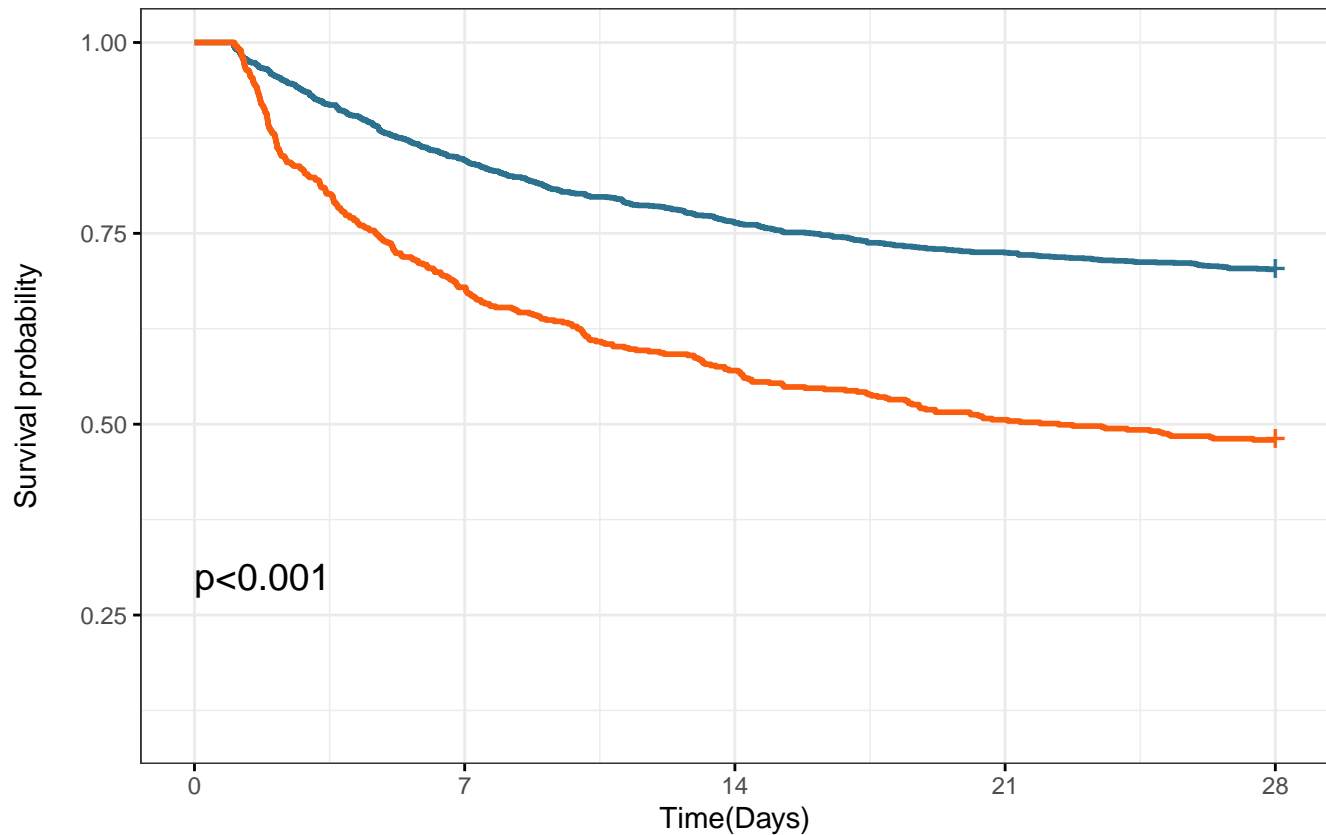

Cumulative number of events

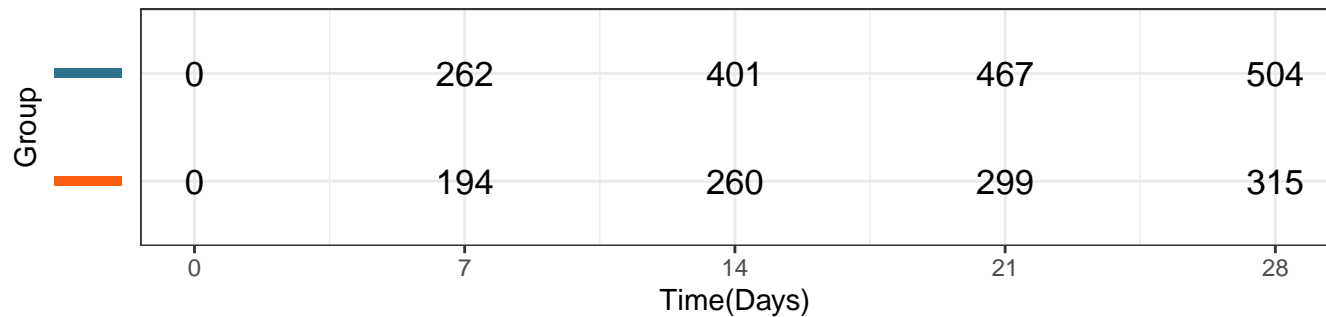

Supplement: Supplementary file 1 — Supporting Information Additional supporting information can be found online in the Supporting Information section. Figure S1 Unadjusted Kaplan–Meier curve for secondary outcome. (A) Kaplan–Meier curve of ICU mortality for the MIMIC‐IV cohort. (B) Kaplan–Meier curve of ICU mortality for the eICU cohort. Figure S2: Unadjusted Kaplan–Meier curve for 28‐day outcome. Figure S3: Feature selection results using the Boruta algorithm. (A) Relative importance of candidate predictors for in‐hospital mortality for the MIMIC‐IV cohort. (B) Relative importance of candidate predictors for in‐hospital mortality for the eICU cohort. Figure S4: Forest plot of subgroup analysis. (A) Forest plot of subgroup analysis of ICU mortality for the MIMIC‐IV cohort. (B) Forest plot of subgroup analysis of ICU mortality for the eICU cohort. Figure S5: Forest plot of subgroup analysis of 28‐day mortality for the MIMIC‐IV cohort. Figure S6: SHAP (SHapley Additive exPlanations) summary plots showing the contribution of the top features to the LightGBM model predictions. (A) SHAP summary plots of the LightGBM model for the MIMIC‐IV cohort. (B) SHAP summary plots of the LightGBM model for the eICU cohort. Figure S7: SHAP (SHapley Additive exPlanations) summary plots showing the contribution of the top features to the XGBoost model predictions. (A) SHAP summary plots of the XGBoost model for the MIMIC‐IV cohort. (B) SHAP summary plots of the XGBoost model for the eICU cohort. Table S1: Basic demographic characteristics of the MIMIC‐IV cohort. Table S2: Basic demographic characteristics of the eICU cohort. Table S3: Unadjusted log‐rank test for in‐hospital mortality of the MIMIC‐IV cohort. Table S4: Unadjusted log‐rank test for ICU mortality of the MIMIC‐IV cohort. Table S5: Unadjusted log‐rank test for 28‐day mortality of the MIMIC‐IV cohort. Table S6: Unadjusted log‐rank test for in‐hospital mortality of the eICU cohort. Table S7: Unadjusted log‐rank test for ICU mortality of the eICU cohort. Table [file CDR-2026-8989505-s001.zip › Sypplementary Figure 2.pdf]

**A**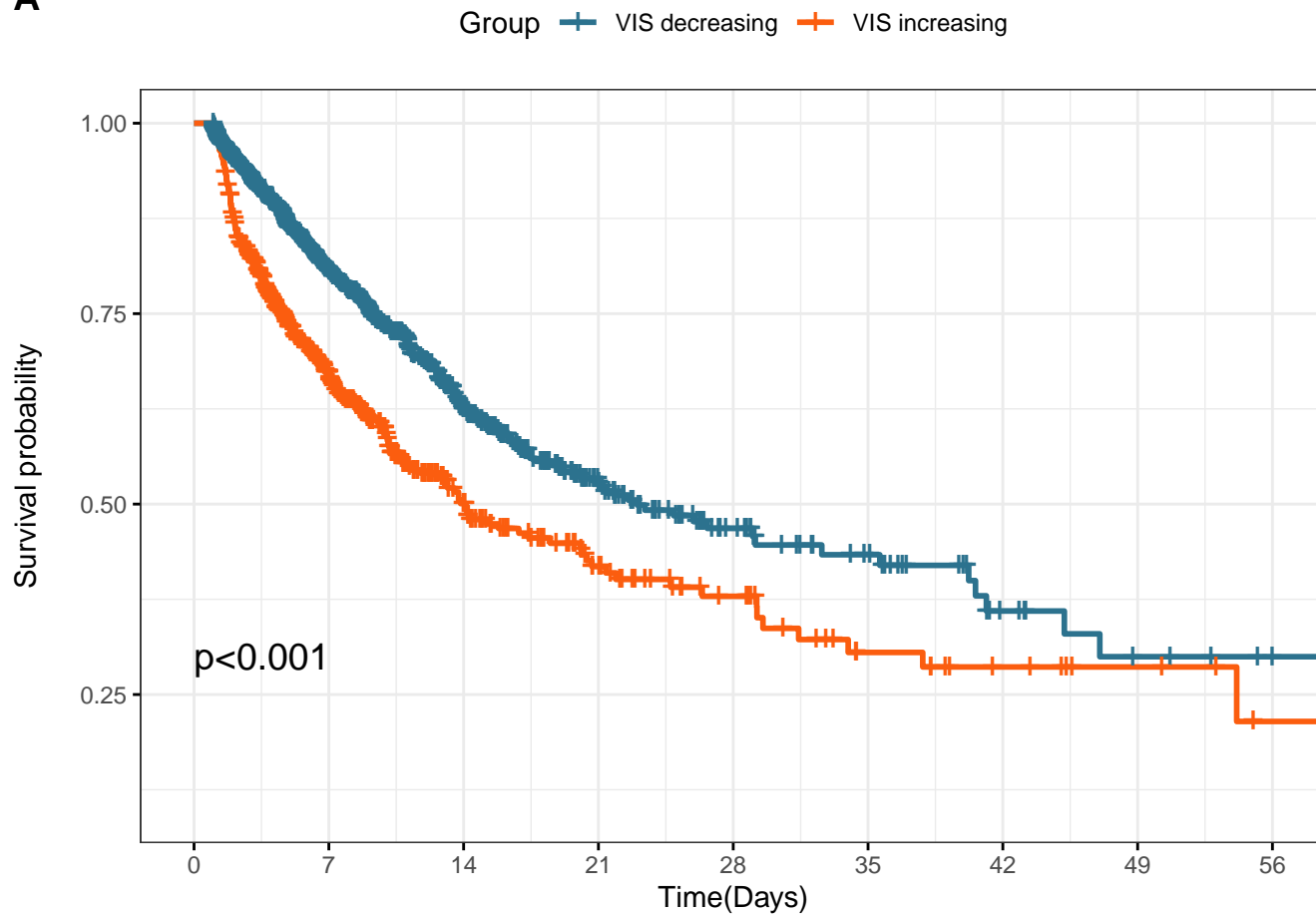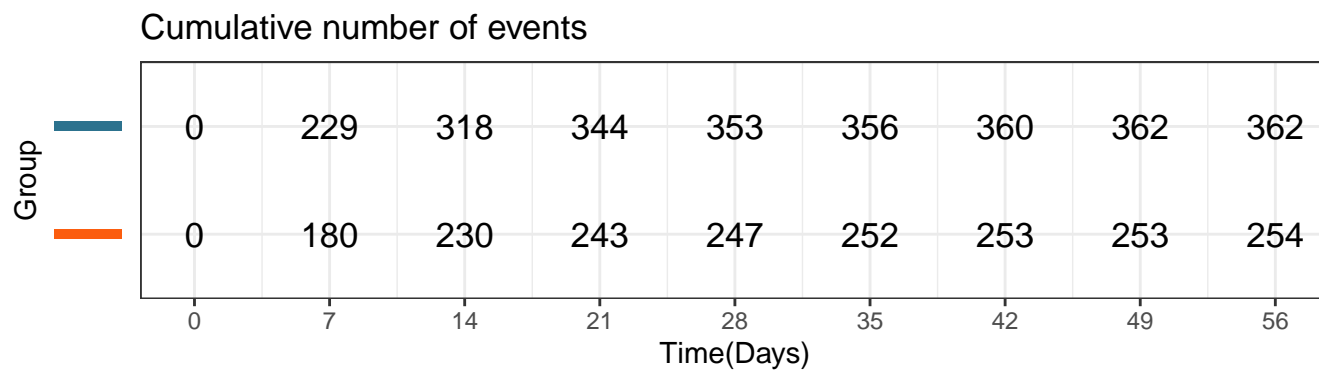**B**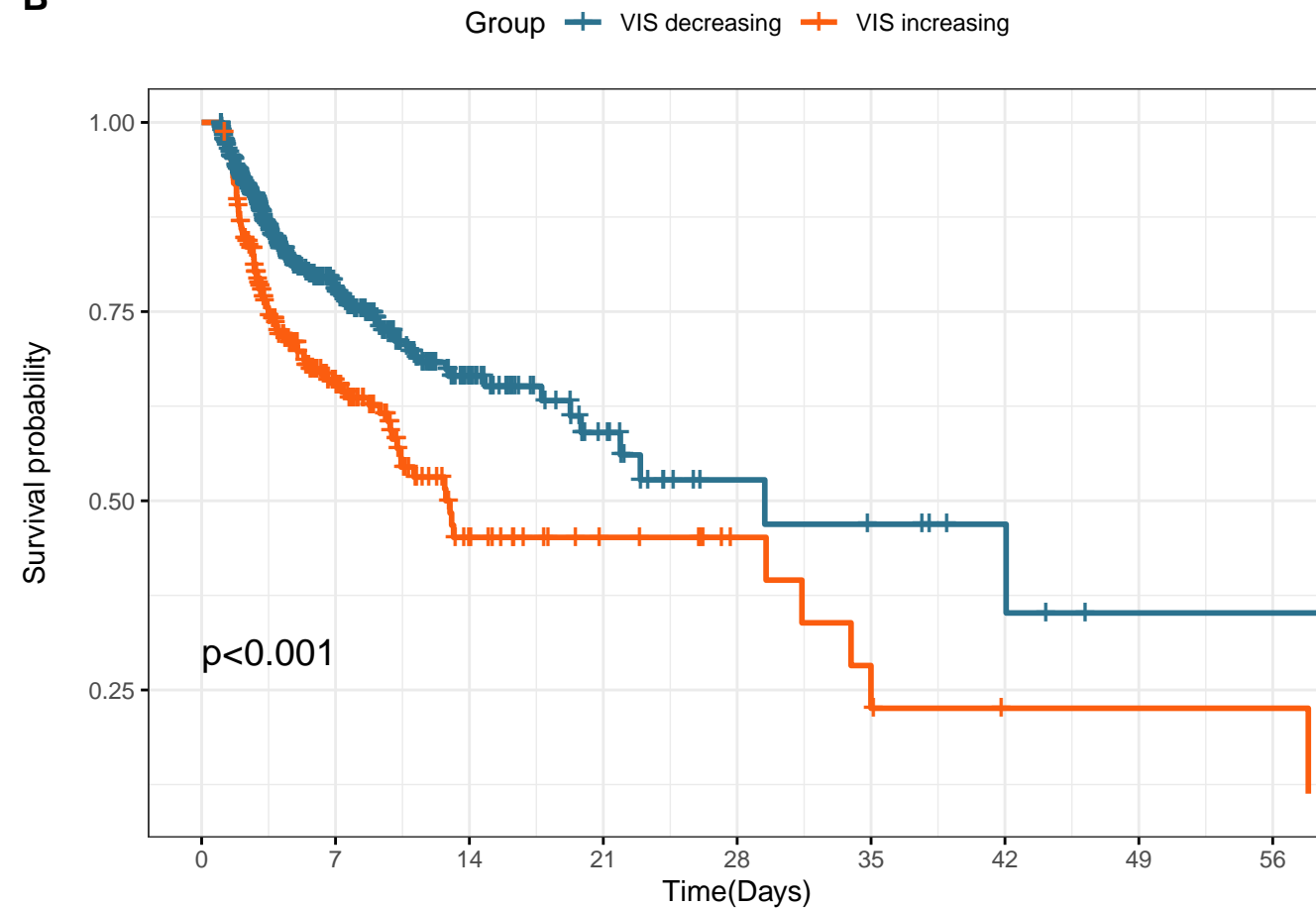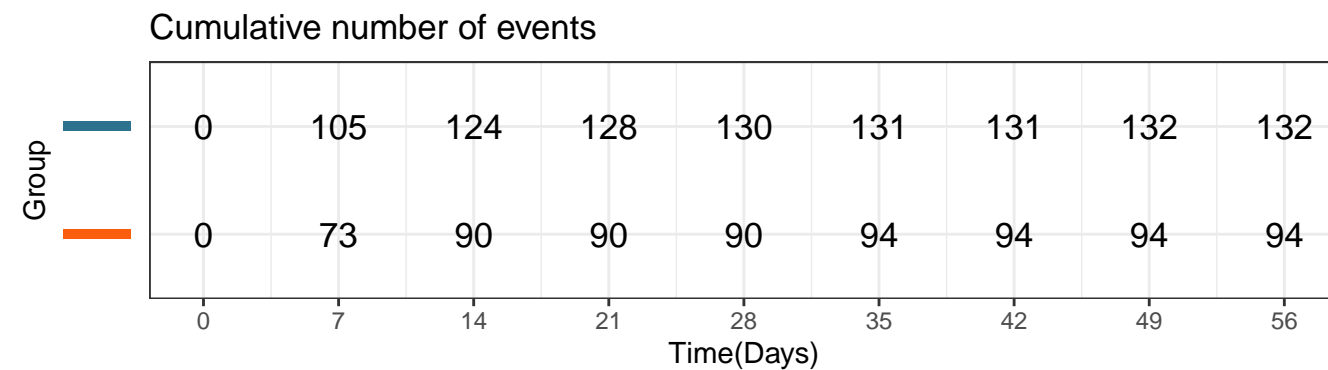

Supplement: Supplementary file 1 — Supporting Information Additional supporting information can be found online in the Supporting Information section. Figure S1 Unadjusted Kaplan–Meier curve for secondary outcome. (A) Kaplan–Meier curve of ICU mortality for the MIMIC‐IV cohort. (B) Kaplan–Meier curve of ICU mortality for the eICU cohort. Figure S2: Unadjusted Kaplan–Meier curve for 28‐day outcome. Figure S3: Feature selection results using the Boruta algorithm. (A) Relative importance of candidate predictors for in‐hospital mortality for the MIMIC‐IV cohort. (B) Relative importance of candidate predictors for in‐hospital mortality for the eICU cohort. Figure S4: Forest plot of subgroup analysis. (A) Forest plot of subgroup analysis of ICU mortality for the MIMIC‐IV cohort. (B) Forest plot of subgroup analysis of ICU mortality for the eICU cohort. Figure S5: Forest plot of subgroup analysis of 28‐day mortality for the MIMIC‐IV cohort. Figure S6: SHAP (SHapley Additive exPlanations) summary plots showing the contribution of the top features to the LightGBM model predictions. (A) SHAP summary plots of the LightGBM model for the MIMIC‐IV cohort. (B) SHAP summary plots of the LightGBM model for the eICU cohort. Figure S7: SHAP (SHapley Additive exPlanations) summary plots showing the contribution of the top features to the XGBoost model predictions. (A) SHAP summary plots of the XGBoost model for the MIMIC‐IV cohort. (B) SHAP summary plots of the XGBoost model for the eICU cohort. Table S1: Basic demographic characteristics of the MIMIC‐IV cohort. Table S2: Basic demographic characteristics of the eICU cohort. Table S3: Unadjusted log‐rank test for in‐hospital mortality of the MIMIC‐IV cohort. Table S4: Unadjusted log‐rank test for ICU mortality of the MIMIC‐IV cohort. Table S5: Unadjusted log‐rank test for 28‐day mortality of the MIMIC‐IV cohort. Table S6: Unadjusted log‐rank test for in‐hospital mortality of the eICU cohort. Table S7: Unadjusted log‐rank test for ICU mortality of the eICU cohort. Table [file CDR-2026-8989505-s001.zip › Sypplementary Figure 1.pdf]
